# Identification of Novel Risk Loci for Common B-Cell Lymphoma Subtypes Through Cross-Trait Analysis with Idiopathic Inflammatory Myopathies

**DOI:** 10.3390/cancers18101536

**Published:** 2026-05-09

**Authors:** Weng Ian Che, James N. Jarvis, International Lymphoma Epidemiology Consortium (InterLymph), IMACS Genetics Group (MYOGEN), Ingrid E. Lundberg, Karin E. Smedby, Janine A. Lamb, Marie Holmqvist

**Affiliations:** 1 Department of Public Health and Medicinal Administration, Faculty of Health Sciences, University of Macau, Macau SAR, China; wengianche@um.edu.mo; 2 Department of Pediatrics and Center for Indigenous Health, University of Washington School of Medicine, Seattle, WA 98195, USA; 3 Division of Rheumatology, Department of Medicine, Solna, Karolinska Institutet, 171 64 Stockholm, Sweden; 4 ME Gastro, Derm and Rheuma, Theme Inflammation and Aging, Karolinska University Hospital, 171 76 Stockholm, Sweden; 5 Clinical Epidemiology Division, Department of Medicine, Solna, Karolinska Institutet, 171 64 Stockholm, Sweden; 6 Hematology Center, Karolinska University Hospital, 171 76 Stockholm, Sweden; 7 Epidemiology and Public Health Group, School of Health Sciences, The University of Manchester, Manchester M13 9PT, UK

**Keywords:** genetic architecture, single nucleotide polymorphism, B-cell lymphomas, diffuse large B-cell lymphoma, follicular lymphoma, chronic lymphocytic leukemia, marginal zone lymphoma

## Abstract

**Objectives:** The genetic architecture of B-cell lymphomas is not fully characterized. We aimed to identify novel candidate variants associated with four common B-cell lymphoma subtypes—diffuse large B-cell lymphoma (DLBCL), follicular lymphoma (FL), chronic lymphocytic leukemia (CLL), and marginal zone lymphoma (MZL)—through cross-trait analyses with correlated idiopathic inflammatory myopathies subtypes, dermatomyositis (DM) and polymyositis (PM). **Methods:** Leveraging shared genetic susceptibility with DM and PM, we applied a pleiotropy-informed conditional false discovery rate (condFDR) method to recompute nominal single nucleotide polymorphism association *p*-values for each B-cell lymphoma subtype. Associations were considered significant at condFDR <0.01. Functional annotation was performed using FUMA (Functional Mapping and Annotation of Genome-Wide Association Studies), followed by Gene Ontology (GO) enrichment analysis via clusterProfiler, with similar GO terms clustered for interpretation and visualization. **Results:** We identified 4, 2, 13, and 6 novel candidate loci for DLBCL, FL, CLL, and MZL, respectively. Most loci exhibited regulatory effects on genes involved in adaptive immune responses and cell death pathways. Notably, for DLBCL locus 1q23.3 affects SLAMF genes implicated in natural killer and lymphocyte activation. A trans-eQTL at 2q13 for FL was associated with BCL11A, a multifunctional oncogene. For CLL, a novel locus at 16q24.1 regulates IRF8, a known CLL risk gene. Functional mapping of previously reported CLL risk loci revealed RIPK1 (6p25.2), linked to necroptosis. No enriched biological pathways were detected for MZL risk-associated genes. **Conclusions:** These findings advance our understanding of the genetic architecture of four B-cell lymphoma subtypes and aim to inform future genetic and functional studies.

## Simple Summary

B-cell lymphomas are a group of blood cancers, but the genetic factors that increase the risk of different types are still not fully understood. In this study, we searched for new genetic signals linked to four common B-cell lymphoma types by taking advantage of their genetic correlation with rare autoimmune muscle diseases known as idiopathic inflammatory myopathies. Using this approach, we discovered several novel genetic variants related to immune regulation and cell death pathways in diffuse large B-cell lymphoma, follicular lymphoma, and chronic lymphocytic leukemia. These discoveries advance our understanding of the genetic basis of common B-cell lymphoma types.

## 1. Introduction

B-cell lymphomas, a major subtype of non-Hodgkin lymphoma (NHL), represent a diverse group of malignancies originating from B lymphocytes at any stage of maturation and differentiation [1,2]. Globally, NHL rank among the 4th to 9th most prevalent cancers in over 90 countries, with average incidence rates of 6.7 and 4.7 per 100,000 person-years in men and women, respectively [3]. Common subtypes include diffuse large B-cell lymphoma (DLBCL), follicular lymphoma (FL), chronic lymphocytic leukemia (CLL), and marginal zone lymphoma (MZL), which can be further classified into more homogeneous subtypes based on tumor site, cellular origin, and genetic aberrations [4,5]. While DLBCL typically presents aggressively, FL, CLL, and MZL are generally indolent but may transform into more aggressive forms, such as DLBCL, over time [1].

The pathogenesis of B-cell lymphomas, like other complex diseases, involves both genetic and environmental factors [1]. Genetic susceptibility is supported by familial aggregation among first-degree relatives [6,7] and the identification of risk-associated variants through genome-wide association studies (GWASs) [8,9,10,11,12,13]. Among the four common B-cell lymphoma subtypes, CLL exhibits the strongest genetic component, with the highest single nucleotide polymorphism (SNP)-based heritability (34%) and the greatest number of identified risk loci (*n* = 40) [14]. Given that common variants typically exert modest effects, large sample sizes are required for their detection. Insufficient sample size may result in missing heritability, where known loci fail to account for the total genetic variance [15]. This phenomenon has been observed for both DLBCL and CLL [14,16].

Idiopathic inflammatory myopathies (IIMs) are rare, clinically diverse diseases unified by muscle inflammation. Dermatomyositis (DM) and polymyositis (PM) are the two broad clinical subtypes traditionally defined based on the Bohan and Peter criteria, with PM lacking the characteristic skin manifestations of DM [17]. Both DM and PM are associated with increased NHL risk near diagnosis and showed suggestive, though statistically non-significant, familial associations with DLBCL and FL based on limited case numbers [18,19,20]. Furthermore, our recent work identified several shared loci in human leukocyte antigen (HLA) and non-HLA regions between these B-cell lymphoma subtypes and DM and PM, along with substantial genetic enrichment of low-*p*-value CLL SNPs when conditioning on low-*p*-value SNPs from DM and PM [21].

Cross-trait analysis using summary statistics from genetically correlated traits offers an efficient alternative to discover novel disease-associated variants when individual-level data or larger cohorts are unavailable. One such approach is the pleiotropy-informed conditional false discovery rate (condFDR), a more statistically efficient multiple testing correction method that leverages genetic information of corrected traits [22]. In this study, we apply condFDR to enhance GWAS discovery of DLBCL, FL, CLL, and MZL by leveraging their genetic pleiotropy with DM and PM.

## 2. Materials and Methods

### 2.1. Data Sources

SNP association summary statistics for four B-cell lymphoma subtypes were obtained from published GWASs, with genotype imputation performed via IMPUTE2 using the 1000 Genomes Project version 3 reference panel [8,9,10,11]. For DLBCL, data were meta-analyzed from four discovery cohorts, comprising 9,116,853 SNPs from 3587 cases and 7666 controls [8]. The FL dataset included 9,078,855 SNPs from 2847 cases and 8107 controls across three discovery and one replication cohorts [9]. For CLL, the meta-analysis incorporated 9,098,434 SNPs from 3100 cases and 7667 controls from four discovery cohorts [11]. The MZL GWAS comprised 8,478,065 SNPs from 825 cases and 6221 controls [10].

For IIM subtypes, summary-level SNP associations were obtained from Zhu et al. using imputed data from previous GWAS and ImmunoChip studies via the Trans-Omics for Precision Medicine Imputation Server [23,24,25]. The GWAS datasets included 8,668,073 SNPs from 402 DM and 255 PM cases and 4336 shared controls, while the ImmunoChip datasets included 1,201,875 SNPs from 870 DM and 923 PM cases and 7486 shared controls.

We included only individuals of European ancestry. Summary statistics were quality controlled using MungeSumstats (version 1.14.1) and aligned to GRCh37 using rtracklayer::liftOver (version 1.24.0; http://master.bioconductor.org/packages/release/workflows/html/liftOver.html, accessed on 4 May 2026) in R (version 4.4.1; https://www.r-project.org) [26,27] (Supplementary Table S1). SNPs that were duplicated, multiallelic, located in conversion-unstable regions, failed liftover, or had missing or zero effect measures were filtered. For DLBCL, FL, and CLL, SNPs from a single-study group were also excluded. We aligned reference alleles to the 1000 Genomes phase 2 reference genome sequence in GRCh37, and Z scores, computed based on *p*-values, were flipped when necessary. SNPs unmatched or missing in the reference genome were filtered out.

Each B-cell lymphoma subtype was paired with GWAS and ImmunoChip data for DM and PM, resulting in eight GWAS and eight ImmunoChip disease pairs. Only SNPs shared between each disease pair were retained for analysis (Supplementary Table S2).

### 2.2. Statistical Methods

To identify novel genetic variants associated with the risks of the four B-cell lymphoma subtypes, we employed pleiotropy-informed condFDR; this approach improves statistical efficiency by leveraging genetic information from a correlated disease to correct for multiple testing of SNP associations for a primary disease [22]. CondFDR offers greater statistical power than standard Bonferroni correction, enabling detection of SNPs that may be moderately associated with the risk of a B-cell lymphoma subtype but did not previously attain genome-wide significance (*p*-value < 5 × 10^−8^) due to limited power. Moreover, this model-free method does not assume any specific pleiotropic mechanism between traits, thereby reducing the risk of bias from model misspecification.

CondFDR values for SNPs of a given B-cell lymphoma subtype were computed using the conditional empirical cumulative distribution function (cdf) of *p*-values of the corresponding SNPs of a given IIM subtype. To reduce the risk of artificially inflated genetic enrichment, we excluded SNPs located in the HLA (25 to 34 Mb) and the chromosome 8p23.1 region from fitting the conditional cdf but kept them in the condFDR analysis. Furthermore, we used intergenic inflation control to adjust nominal *p*-values of SNPs. We defined statistical significance as condFDR < 0.01, a conservative threshold chosen to minimize the risk of false positive findings [22]. SNPs passing this threshold were clumped (r^2^ < 0.1) in linkage disequilibrium (LD) windows of 10,000 kb, and loci within 250 kb were merged.

All identified lead SNPs and their LD proxy SNPs (r^2^ > 0.8) except those in the extended HLA region were positionally and functionally mapped to protein-coding genes using FUMA (Functional Mapping and Annotation of Genome-Wide Association Studies) SNP2GENE [28]. Functional annotation included *cis*-acting (± 1 Mb) and *trans*-acting (>1 Mb) expression quantitative trait locus (*cis/trans*-eQTL) and Hi-C chromatin interaction analysis, restricted to data from whole blood, CD19+ B cells, lymphoblastoid cell lines (LCLs), and peripheral blood mononuclear cells (PBMC). In eQTL mapping, the regulatory direction (upregulation “+” or downregulation “−”) was inferred from the sign of the test statistics for the tested allele. If the tested allele did not correspond to the risk-increasing allele in the input data, the test statistic’s sign was flipped. Chromatin interaction mapping included only enhancer-promoter interactions where enhancer regions overlapped the tested SNP and the interacting regions overlapped promoter regions (defined as 250 bp up- and 500 bp downstream of the transcription start site) of protein-coding genes. Enhancer and promoter annotations were done using the Roadmap Epigenomics Projects for 111 epigenomes [29]. Significant associations were defined as eQTLs with FDR <0.05 and chromatin interactions with FDR <1 × 10^−6^. Parameter details are provided in Supplementary Table S3.

For each B-cell lymphoma subtype, mapped genes were subjected to Gene Ontology (GO) enrichment analysis (biological process only) using clusterProfiler (version 4.14.6) and the org.Hs.eg.db (version 3.20.0) annotation packages [26,30]. Significance was defined as an FDR <0.05, and significantly enriched GO terms were simplified using semantic similarity-based clustering (threshold = 0.6).

This study was approved by the Swedish Ethical Review Authority (reference numbers 2020-04529 and 2023-01343-02), with approvals granted on 12 October 2020 and 9 March 2023, respectively.

## 3. Results

By conditioning on SNP *p*-values from the GWAS and ImmunoChip datasets of DM and PM, we identified 11, 8, 39, and 9 loci (condFDR < 0.01) associated with the risk of DLBCL, FL, CLL, and MZL, respectively (Supplementary Tables S4–S7). Of these, 4, 2, 13, and 6 had not been previously reported at genome-wide significance and are proposed as novel risk loci (Table 1). All loci within the extended HLA region were previously reported except rs10946876 (6p22.2) for CLL. Functional mapping findings are presented by B-cell lymphoma subtype. eQTL and chromatin interaction findings were derived from whole blood, CD19+ B cells, LCLs, or PBMC. This information will not be reiterated in the following section for simplicity.

### 3.1. DLBCL Risk-Associated Loci

**Figure 1:**
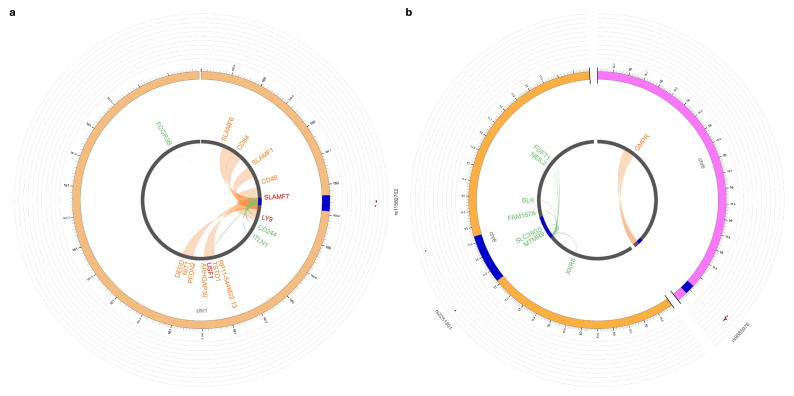
eQTLs and Hi-C chromatin interactions at novel DLBCL risk loci. (**a**) Chromosome 1. (**b**) Chromosomes 6 and 8 (arranged clockwise). The outermost layer is the Manhattan plot displaying the lead SNPs, with their respective SNP IDs labeled. The outer circle is the chromosome coordinate with genomic risk loci highlighted in blue. Genes mapped by either eQTLs (green), chromatin interaction (orange), or both (red) are shown on the inner circle. eQTL and chromatin interaction links are shown as links in green and orange, respectively. DLBCL: diffuse large B-cell lymphoma; SNP: single nucleotide polymorphism; eQTL: expression quantitative trait locus.

Of the four novel loci associated with DLBCL risk, three showed regulatory effects on gene expression (Figure 1 and Supplementary Tables S8 and S9). Specifically, our analysis showed that rs11582702 (1q23.3) acts as a *cis*-eQTL for several signaling lymphocytic activation molecule receptor family (SLAMF) genes, including *SLAMF7*, *LY9*, and *CD244* (Figure 1a). Furthermore, the variant was associated with both increased and decreased expression across different whole blood datasets. It also upregulates *ITLN1* and *USF1* and affects *FCGR3B*, as well as overlapping enhancer regions interacting with promoters of multiple SLAMF receptor genes and others. Notably, the genomic region encompassing *SLAMF1* has previously been reported to confer IIM risk in aggregate association testing [31]. rs9885976 (6p22.3) was implicated in the regulation of *GMPR* via an enhancer-promoter interaction (Figure 1b). rs2251301 (8p23.1), located within *FAM167A*, regulates its expression (+/−) and affects expression of six additional nearby genes: *XKR6* (+), *MTMR9* (−), *SLC35G5* (+), *BLK* (+), *NEIL2* (−), and *FDFT1* (−). Variants in the locus 8p23 positionally mapped to *BLK* have been linked with DM and PM risks [24,32,33,34]. No regulatory function was identified for rs117408955 (13q34).

Among six previously reported DLBCL risk loci, four demonstrated regulatory activity through *cis*-eQTL effects or enhancer-promoter interactions. Specifically, these loci affected *NCOA1*, *DNAJC27*, *POMC* (rs79480871, 2p23.3); *EOMES*, and *CMC1* (rs6795177, 3p24.1); *POLQ*, *GOLGB1*, *IQCB1*, *EAF2*, *ILDR1*, *CCDC58*, and *FAM162A* (rs9831894, 3q13.33); and *MYC* (rs13255292, 8q24.21) (Supplementary Figure S1, Tables S10 and S11).

**Figure 2:**
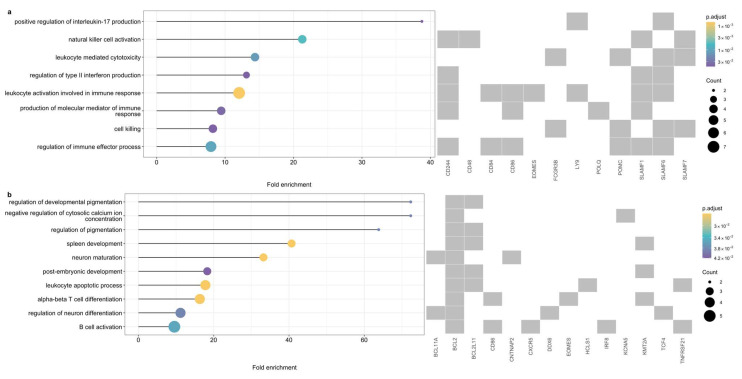
Significantly enriched Gene Ontology biological processes (false discovery rate < 0.05) among genes mapped in: (**a**) diffuse large B-cell lymphoma and (**b**) follicular lymphoma.

In total, 41 genes were positionally or functionally mapped to the 10 DLBCL risk loci. Eight gene sets comprising 12 unique genes were significantly enriched in 28 biological processes (simplified to 8), including inflammatory responses (e.g., interleukin-17 and type II interferon production) and innate and adaptive immune activity (e.g., natural killer (NK) cell/leukocyte activation and leukocyte-mediated cytotoxicity) (Figure 2a and Supplementary Table S12). Of note, all enriched processes involved at least two SLAMF receptor genes.

### 3.2. FL Risk-Associated Loci

**Figure 3:**
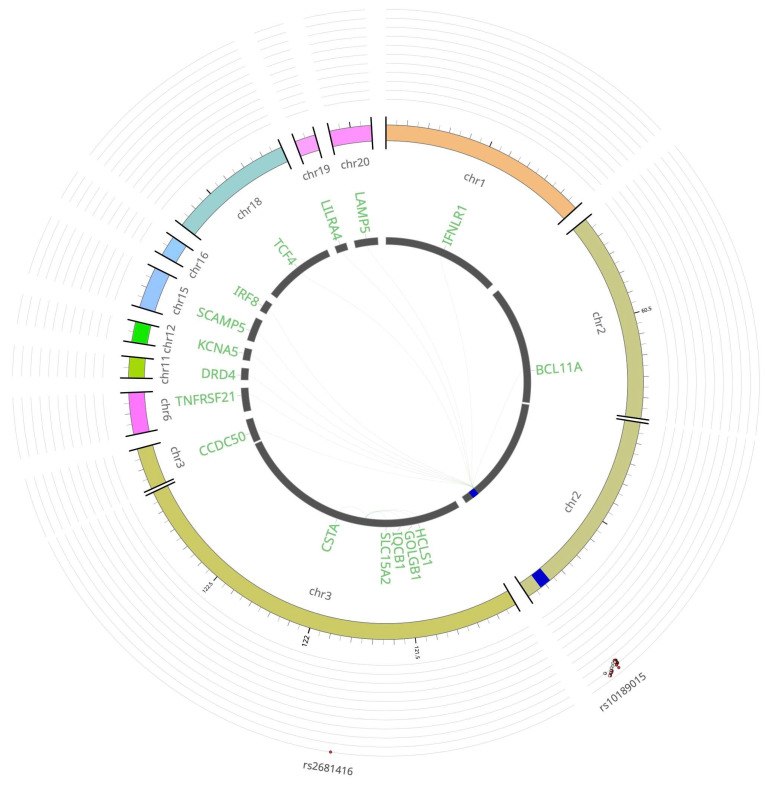
eQTLs at FL risk loci. Chromosomes 1, 2, 3, 6, 11, 12, 15, 16, 18, 19, and 20 (arranged clockwise). The outermost layer is the Manhattan plot displaying the lead SNPs, with their respective SNP IDs labeled. The outer circle is the chromosome coordinate with genomic risk loci highlighted in blue. Genes mapped by *cis*/*trans*-eQTLs are shown on the inner circle. *Cis*/*trans*-eQTL links are shown as links in green. FL: follicular lymphoma; SNP: single nucleotide polymorphism; eQTL: expression quantitative trait locus.

Among the two novel FL risk loci, the LD proxy rs1439287 (r^2^ > 0.98) of rs10189015 (2q13) functions as a *trans*-eQTL. It upregulates *BCL11A* (>51 Mb upstream) and multiple genes across different chromosomes, including *IFNLR1* (chr1), *CCDC50* (chr3), *TNFRSF21* (chr6), *DRD4* (chr11), *KCNA5* (chr12), *SCAMP5* (chr15), *IRF8* (chr16), *TCF4* (chr18), *LILRA4* (ch19), and *LAMP5* (chr20) (Figure 3 and Supplementary Table S13). Notably, recurrent somatic mutation of *IRF8* has been observed in 6% of FL cases [35]. The locus rs2681416 (3q13.33) acts as a *cis*-eQTL for *HCLS1* (−), *GOLGB1* (+), *IQCB1* (+), *SLC15A2* (−), and *CSTA* (+). Although mapped to different loci, variants at 11p15.5 (*DRD4*) and 16q24.1 (*IRF8*) have been linked to IIM in a GWAS meta-analysis of seropositive rheumatic diseases [34].

Of the five previously reported FL risk loci, rs2887944 (3p24.1), rs13254990 (8q24.21), rs10892296 (11q23.3), and rs17749561 (18q21.33), along with their LD proxies, showed regulatory effects on nearby genes (Supplementary Figure S2, Tables S14 and S15). These included *EOMES*, *CMC1*, *AZI2*, *ZCWPW2; MYC*; *ATP5MG*, *KMT2A*, *IFT46*, *ARCN1*, *DDX6*, *CXCR5*, and *TRAPPC4*; and *BCL2*, *KDSR*, and *VPS4B*. The 3p24.1 locus has previously shown a suggestive association with IIM risk [34]. Additionally, rs4245081 (11q24.3) was linked to an enhancer-promoter interaction involving *FLI1* and regulates *CNTNAP2* (+, chr7), *MOB3B* (+, chr9), and TTC16 (−, chr9).

Genes mapped to the eight FL risk-associated loci were significantly enriched in 21 biological processes (simplified to 10), including regulation of pigmentation, negative regulation of cytosolic Ca^2+^ concentration, spleen development, neuron maturation and differentiation, leukocyte apoptotic process, alpha-beta T-cell differentiation, and B-cell activation (Figure 2b and Supplementary Table S16).

### 3.3. CLL Risk-Associated Loci

**Figure 4:**
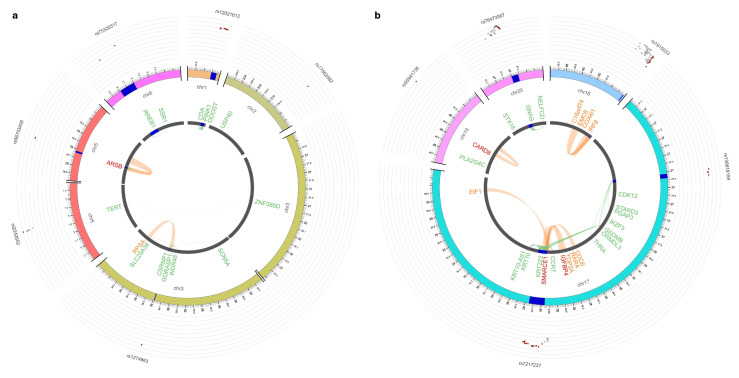
eQTLs and Hi-C chromatin interactions at CLL risk loci. (**a**) Chromosomes 1, 2, 3, 5, and 6. (**b**) Chromosomes 16, 17, 19, and 20 (arranged clockwise). The outermost layer is the Manhattan plot displaying the lead SNPs, with their respective SNP IDs labeled. The outer circle is the chromosome coordinate with genomic risk loci highlighted in blue. Genes mapped by either *cis*/*trans*-eQTLs (green), chromatin interaction (orange), or both (red) are shown on the inner circle. *Cis*/*trans*-eQTL and chromatin interaction links are shown as links in green and orange, respectively. CLL: chronic lymphocytic leukemia; SNP: single nucleotide polymorphism; eQTL: expression quantitative trait locus.

Among the 13 novel CLL risk loci, 11 showed regulatory activity via *cis*- or *trans*-eQTLs or enhancer-promoter interactions, affecting 41 genes (Figure 4 and Supplementary Tables S17 and S18). *IKZF3*, one of the mapped genes, is mutated in 1–2% of CLL cases [35]. Among other mapped genes, *ARSB* (5q14.1), *SMARCE1* (17q21.2), and *CARD8* (19q13.33) were supported by both eQTL and chromatin interaction findings. Notably, two regulatory loci novel to CLL (16q24.1 and 17q12) have been previously associated with IIM risk [32,34]; similarly, *EOMES* has been linked to IIM risk, albeit via a different locus at 3p24.1 [34].

Of the 26 previously reported CLL risk loci, 25 showed regulatory effects on 90 nearby genes (Supplementary Figure S3, Tables S19 and S20). These included key B-cell lymphoma oncogenes such as *BCL2*, *MYC*, and *BCL6*, as well as *UBR5*, *RIPK1*, *PPIL3*, *CFLAR*, *TRAK2*, *SLC16A14*, *SP140L*, *SP100*, *CAB39*, *LINC03047*, *STAMBPL1*, *ACTA2*, *FAS*, *RFX7*, *MNS1*, and *PMAIP*, with regulatory evidence from both eQTL and Hi-C data. Several loci also act as *trans*-eQTLs, regulating 30 genes on different chromosomes. Although rare (<1%), somatic mutations in *BCL2*, *MYC*, and *UBR5* have been reported in CLL [35].

**Figure 5:**
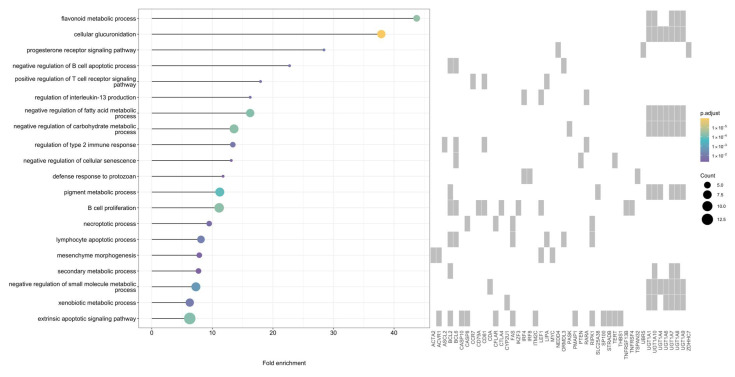
Top 20 significantly enriched Gene Ontology biological processes (false discovery rate < 0.05) among genes mapped in chronic lymphocytic leukemia.

In total, 86 genes associated with CLL risk loci were significantly enriched in 133 biological processes (simplified to 37), many of which were related to B-cell apoptosis and immune regulation and signaling pathways (Figure 5, Supplementary Figure S4 and Table S21).

### 3.4. MZL Risk-Associated Loci

**Figure 6:**
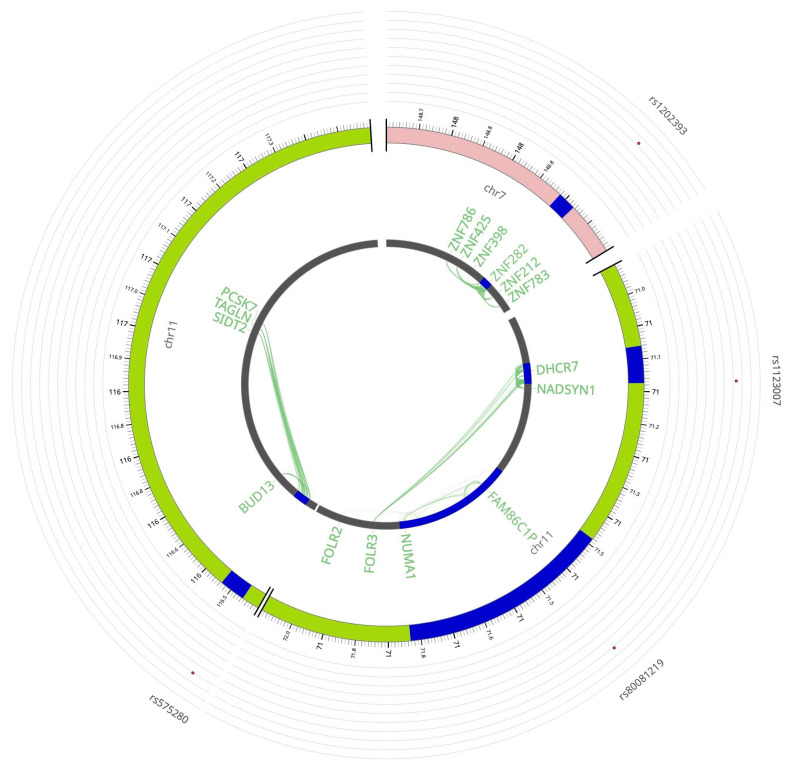
eQTLs and Hi-C chromatin interactions at MZL risk loci on chromosomes 7 and 11 (arranged clockwise). The outermost layer is the Manhattan plot displaying the lead SNPs, with their respective SNP IDs labeled. The outer circle is the chromosome coordinate with genomic risk loci highlighted in blue. Genes mapped by either *cis*/*trans*-eQTLs (green), chromatin interaction (orange), or both (red) are shown on the inner circle. *Cis*/*trans*-eQTL and chromatin interaction links are shown as links in green and orange, respectively. MZL: marginal zone lymphoma; SNP: single nucleotide polymorphism; eQTL: expression quantitative trait locus.

Of the six novel MZL risk loci, four act as *cis*-eQTLs at 7q36.1 (*ZNF786*, *ZNF425*, *ZNF282*, *ZNF212*, *ZNF783*, and *ZNF398*), 11q13.4 (*FOLR3*, *DHCR7*, *NADSYN1I*, *FOLR3*, *FOLR2*, *FAM86C1P*, and *NUMA1*), and 11q23.3 (*BUD13*, *PCSK7*, *SIDT2*, and *TAGLN*) (Figure 6 and Supplementary Table S22).

Among the three previously reported MZL risk loci, only rs190777845 (6p21.31) showed regulatory activity as a *cis*-eQTL regulating *FGD2* (+), *ETV7* (−) and mediating *SRSF3* through enhancer and promoter interaction (Supplementary Figure S5, Tables S23 and S24).

No GO-defined biological processes were significantly enriched among the MZL risk-associated genes.

## 4. Discussion

By leveraging genetic pleiotropy with DM and PM, we identified 4, 2, 13, and 6 novel risk loci for DLBCL, FL, CLL, and MZL, respectively. This yield significantly exceeded that of a conventional meta-analysis across FL, CLL, and MZL, which identified only one novel genome-wide significant locus, rs11187157 at 10q23.33, primarily driven by CLL [36]. Without increasing sample size, our pleiotropy-informed condFDR analyses identified the same locus for CLL and revealed its regulatory functions on *TNKS2* (0.9 Mb upstream), *TCF7* on chromosome 5, and *CLEC4E* on chromosome 12, in addition to the previously reported *HHEX* [36]. Furthermore, a recent pleiotropy-informed multi-trait GWAS across eight lymphoid malignancy subtypes identified 20 novel loci with genome-wide significance, three of which (rs2887944 at 3p24.1 for FL, rs34517439 at 1p31.1, and rs142239370 at 10q22.1 for CLL) overlapped with our findings [37]. The same study also reported rs28876421 at 3q13.33 for lymphoid and B-cell-derived malignancies. Within this locus, we identified an independent SNP, rs2681416 (r2 = 0.16 in European populations), associated with FL risk. Additionally, the largest GWAS meta-analysis of FL, presented in a recent conference abstract, also reported associations at 2q13 (*BCL2L11*) and 3q13 (*CD86*) loci [38]. Collectively, these results underscore the effectiveness of pleiotropy-informed condFDR in uncovering novel disease-risk-associated loci and contribute to narrowing the gap in missing heritability of B-cell lymphomas [14,16].

Using extensive eQTL and Hi-C datasets, we revealed regulatory functions at both novel and established B-cell lymphoma loci, many of which were not functionally mapped until the work of Guler and Canzian [8,9,10,11,14,36,37,39,40,41,42,43]. These variants regulate the expression of proximal and distal genes—including inter-chromosomal targets—implicated in immune regulation and signaling pathways. Among the novel loci identified in our study, two showed regulatory functions on genes recurrently mutated in DLBCL, FL, and CLL. The FL-associated variant rs10189015 at 2q13 that acts as a *trans*-eQTL for *IRF8*. *IRF8*, involved in B-cell activation, has been found to be recurrently mutated in indolent FL (6%), transformed FL (tFL) (9%), and DLBCL (5%) [35]. Another novel variant, rs139818184 at 17q12, associated with CLL, is a *cis*-eQTL downregulating *IKZF3* in whole blood. *IKZF3*, involved in B-cell development, has been found to be mutated in 1–2% in CLL and 5% of DLBCL cases [35].

Our functional mapping of previously reported loci aligns closely with findings of Guler and Canzian, who fine-mapped lymphoma risk loci and performed functional annotation using *cis*-molecular QTL data [37]. Incorporating *trans*-eQTL and Hi-C data, we identified additional candidate effector genes. These included *POLQ* (18q21.33) for DLBCL; *KMT2A* (11q23.3) for FL; *CFLAR* (2q33.1), *SP100* (2q37.1), *RIPK1* (6p25.3), *TNKS2*, and *TCF7* (10q23.33) for CLL. These genes are involved in immune-related, cell death, and Wnt signaling pathways, discussed in detail below.

Somatic genetic aberrations in B-cell lymphomas are critical for understanding pathogenesis, classification, prognosis, and therapeutic responses [35]. However, a power analysis of approximately 5000 paired tumor-normal samples across 21 cancer types indicated that >2000 and >500 pairs are required to detect 90% of oncogenes mutated at ≤2% above the background mutation frequency for DLBCL and CLL, respectively [44]. Current large sequencing studies fall short of these thresholds [45,46], suggesting that many low-frequency mutations remained undetected. Although somatic mutations can arise independently of germline mutations, the regulatory loci identified here may guide targeted sequencing efforts to uncover novel rare somatic mutations for DLBCL, FL, CLL, and MZL, as well as inform functional studies of their biological impact.

Analysis of genes mapped to the novel and previously reported genome-wide significant loci for DLBCL, FL, and CLL revealed significant enrichment in diverse biological processes, particularly those related to immune function. Genes associated with novel loci comprised 67%, 43%, and 33% of the total mapped genes for DLBCL, FL, and CLL, respectively. In DLBCL, enrichment in NK cell and lymphocyte activation pathways was predominantly driven by genes encoding SLAMF receptors. These receptors, part of the CD2 immunoglobulin superfamily, are broadly expressed in hematopoietic cells including NK, T, and B cells, and are differentially expressed in B-cell lymphomas [47]. Some SLAMF receptor expression has been linked to the prognosis and treatment response in DLBCL. For instance, elevated SLAMF1 expression in Farage cells (an Epstein–Barr virus+ DLBCL cell line) correlated with chemotherapy resistance, potentially due to its ability to enhance cell survival [48,49]. Conversely, SLAMF2 upregulation was associated with better survival, likely through enhanced NK and T cell cytotoxicity [50,51]. While SLAMF3, SLAMF5, and SLAMF6 are expressed in DLBCL, their roles in DLBCL pathogenesis and clinical outcomes remain unclear [47]. Given their immunomodulatory functions, SLAMF receptors are emerging targets for immunotherapy against multiple myeloma, including monoclonal antibodies and chimeric antigen receptor T-/NK cell products [52]. Our findings of genetic susceptibility affecting expression of SLAMF receptors in DLBCL not only implicate NK and cytotoxic T cells in disease pathogenesis but also underscore the pathological relevance of SLAMF receptors as potential therapeutic targets.

For FL, we found significant enrichment in pathways related to spleen development (*BCL2*, *BCL2L11*, and *KMT2A*), leukocyte apoptosis (*BCL2* and *BCL2L11*), T-cell differentiation (BCL2, *CD86*, *EOMES*, and *KMT2A*), and B-cell activation (*BCL2*, *CD86*, *CXCR5*, *IRF8*, and *TNFRSF21*). These findings align with known FL pathogenesis, including *BCL2* overexpression, skewed CD4+ T-cell distribution in the tumor microenvironment, and constitutive B-cell receptor signaling activation [53]. Notably, *KMT2D* and *TNFRSF14*—frequently mutated in FL and tFL—contribute to lymphomagenesis via promoting B-cell activation [35]. Similarly, *KMT2A* and *TNFRSF21*, which are members of the same functional classes, may drive FL pathogenesis through analogous mechanisms. Additional enriched pathways included pigmentation, neuron and post-embryonic development, and cytosolic calcium ion level regulation. These were likely attributable to a multifunctional gene (*BCL11A*) and apoptosis-related genes (*BCL2* and *BCL2L11*), which are broadly involved in tissue development [54,55], rather than FL-specific pathology. Nevertheless, *BCL11A*, an oncogene, has been found to be overexpressed in various malignancies [55]. In CLL, this overexpression has been linked to the t(2;14)(p13;q32.3) translocation [56]. Other genes involved in FL-enriched pathways, including *TCF4*, *HCLS1*, and *DDX6*, have been implicated in DLBCL, CLL, and MZL, respectively [57,58,59]. Further investigation is needed to explore their roles in FL pathogenesis.

The annotated CLL risk-associated genes are implicated in numerous immune regulatory and signaling pathways, aligning with pathological evidence of disrupted B-cell apoptosis and immune dysfunction in the tumor microenvironment [60,61]. Although the mechanisms by which B-cell death dysregulation drives CLL pathogenesis remain unclear, current understanding primarily centers on the intrinsic apoptotic pathway [61]. Our findings reveal enrichment of extrinsic apoptotic and necroptotic pathways involving *FAS*, *CAPS8*, and *RIPK1*. This suggests that additional cell death mechanisms may contribute to B-cell expansion in CLL. This is consistent with reported downregulation of core necroptotic components in CLL [62]. Receptor-interacting protein kinase 1, encoded by *RIPK1*, is a key regulator of the tumor-necrosis factor-induced death receptor signaling pathway. It can promote apoptosis via Caspase-8 (encoded by *CASP8*) activation and necroptosis when Caspase-8 is inhibited. Necroptosis has been proposed as a tumor-suppressive mechanism [63]. Our identified loci demonstrate bidirectional regulation of *CASP8* and downregulation of *RIPK1* in whole blood and B cells. These findings suggest that both RIPK1-mediated apoptosis and necroptosis may potentially be dysregulated, thereby facilitating CLL B-cell survival and progression. Further functional studies are warranted to validate the roles of RIPK1, Caspase-8, and related receptors and regulators such as those encoded by *FAS* and *CFLAR* in CLL lymphomagenesis.

The NF-κB and WNT signaling pathways, both implicated in promoting B-cell survival, were significantly enriched in CLL. This is consistent with their constitutive activation in the disease [61,64]. Somatic mutations in canonical WNT pathway genes, such as *WNT1* and *CREBBP*, have been previously reported in CLL [64]. Our study extends these findings by identifying regulatory loci in *LEF1* and *TCF7*, *UBR5*, *PTEN*, *TNKS2*, *HHEX*, *IGFBP4,* and *WNT3*, all of which encode transcription factors or regulators within the pathway.

Additional annotated pathways related to cell–cell and cell–matrix adhesion may provide insight into the CLL tumor microenvironment, where communications with immune and stromal cells via adhesion are critical for tumor evolution [60]. Xenobiotic metabolism pathways, particularly those involving *UGT1A* genes, were also significantly enriched in CLL. While *UGT1A1* polymorphism has been implicated in CLL pathogenesis [65], our identification of *UGT1A* genes was based solely on genomic proximity, warranting further investigation to confirm their functional relevance.

Although we identified multiple genes regulated by MZL risk loci, their roles in MZL lymphomagenesis remain to be elucidated; no biological pathways were significantly enriched with these annotated genes, and none of them are involved in known pathological pathways of MZL. Further studies are needed to validate these findings.

As noted in the Results, several novel loci identified for DLBCL (1q23.3 and 8p23), FL (11p15.5 and 16q24.1), and CLL (16q24.1 and 17q12) or their mapped genes have previously been associated with IIM risk or implicated in IIM pathogenesis [24,32,33,34]. Many of these genes—including *SLAMF1*, *BLK*, *IRF8*, and *EOMES*—are immune-related. Together, these findings further support dysregulated immunity as a shared pathologic mechanism underlying both B-cell lymphoma and IIM.

As demonstrated in prior research and our present study [36], genetic susceptibility to DLBCL, FL, and CLL is markedly heterogeneous, with partial overlap. Among the genes mapped to our identified novel loci, only *IRF8* was associated with more than one B-cell lymphoma subtype (FL and CLL), and both have also been linked to DLBCL risk [36]. Furthermore, novel regulatory loci identified for FL revealed shared genetic architecture involving *TCF4*, *CMC1*, *AZI2*, *ZCWPW2*, *GOLGB1*, and *IQCB1*. These genes have been functionally mapped to previously reported genome-wide significant loci for DLBCL and CLL [8,11,36,39,40,41,66]. Furthermore, all identified novel loci for MZL were disease-specific, despite prior analyses indicating significant strong genetic correlations between CLL and MZL (correlation coefficient = 0.7, standard error = 0.3). The relatively large standard error, likely reflecting the small MZL sample size, indicates substantial uncertainty in this estimate. Moreover, disparities in GWAS sample sizes across B-cell lymphoma subtypes—particularly the limited MZL cohort—may have contributed to differences in locus discovery in our study.

Genetic associations in this study were identified using a pleiotropy-informed method and therefore warrant replication in future B-cell lymphoma GWAS with larger sample sizes. As most identified variants reached suggestive significance in the original GWAS (*p* < 2.25 × 10^−5^), many loci are likely to be validated as the sample sizes increase in ongoing and future GWAS efforts. Functional validation is also required, with priority given to the variants supported by eQTL and/or Hi-C data and linked to the genes involved in pathways related to B-cell lymphoma pathogenesis (e.g., *SLAMF* genes for DLBCL, *BCL11A* for FL, and caspase genes for CLL). Because functional annotations were derived from the noncancerous cells/tissues, regulatory effects specific to the tumor microenvironment or disease states may not be fully captured. Thus, functional interpretations should be considered provisional. Future studies incorporating lymphoma-specific and tumor-microenvironment-resolved functional genomics data will be essential to more accurately contextualize these findings.

Several mechanisms have been proposed to explain *trans*-eQTL effects, most notably mediation through *cis*-eGenes or transcription factors [67,68]. Although eQTL colocalization analyses can help elucidate these mechanisms, such analyses were not feasible in our study due to the limited statistical power of the original B-cell lymphoma GWAS.

Due to differences in allele frequencies and LD structure across ethnic groups, our findings may primarily apply to populations with European ancestry. Nonetheless, some of our identified novel loci may inform the risk variant discovery in non-European populations. Because several DLBCL-risk loci including 6p25.3, 6p21.3, 8q24.21, 3p24.1, and 3q13.33 have been reported in both European and Chinese populations [8,12,39,69]. A further limitation is the broad subtype classification in InterLymph data, which may obscure the subtype-specific associations within each B-cell lymphoma subgroup [8,9,10,11].

## 5. Conclusions

This study advances our understanding of the genetic architecture of DLBCL, FL, CLL, and MZL by identifying numerous novel loci and functionally annotating both novel and previously reported genome-wide significant loci to the genes implicated in lymphomagenesis, thereby providing a stronger foundation for future etiological and functional studies.

## Figures and Tables

**Table 1:** Novel lead SNPs associated with the risk of four B-cell lymphoma subtypes, identified through analyses conditioned on dermatomyositis and polymyositis.

B-Cell Lymphoma Subtype	SNP ID	CHR:BP in hg19	Function	Reference Allele	Effect Allele	Z Score	*p*-Value	CondFDR	Mapped Protein-Coding Genes §
DLBCL	rs11582702	1:160,727,986	intergenic	C	T	−4.57	6.09 × 10^−6^	0.007251	*SLAMF7*
	rs9885976	6:16,940,672	intergenic	C	T	−4.59	5.50 × 10^−6^	0.005573	
	rs2251301	8:11,119,037	ncRNA_intronic	C	T	−4.93	1.05 × 10^−6^	0.009425	*FAM167A*
	rs117408955	13:112,538,767	intergenic	G	A	−4.32	1.85 × 10^−5^	0.008798	
FL	rs10189015	2:111,918,472	intronic	G	A	5.256825	2.31 × 10^−7^	0.001103	***BCL2L11*** **, ** *ACOXL*
	rs2681416	3:121,817,613	intronic	G	A	5.425597	9.36 × 10^−8^	0.000638	***CD86***
CLL	rs12021613	1:20,961,950	intronic	C	T	−4.86479	1.95 × 10^−6^	0.002663	***PINK1***, *CDA*, *DDOST*
	rs17862882	2:234,682,496	downstream	C	A	4.358045	2.02 × 10^−5^	0.008728	*UGT1A1*, *UGT1A3*, *UGT1A4*, *UGT1A5*, *UGT1A6*, *UGT1A7*, *UGT1A8*, *UGT1A9*, *UGT1A10*, *MROH2A*
	rs1274963	3:39,191,029	intronic	A	G	−4.8292	1.56 × 10^−6^	0.006201	***CSRNP1***
	rs2242652	5:1,280,028	intronic	G	A	5.323386	1.92 × 10^−7^	0.000275	***TERT***
	rs56703405	5:78,186,110	intronic	T	G	4.626346	6.03 × 10^−6^	0.006574	***ARSB***
	rs75320317	6:7,202,278	intronic	T	C	−4.49139	7.90 × 10^−6^	0.008553	***RREB1***
	rs10946876	6:26,896,889	intronic	G	A	4.912766	1.54 × 10^−6^	0.001625	
	rs67063178	16:82,893,008	UTR3	C	T	−4.89011	1.15 × 10^−6^	0.00518	***CDH13***
	rs1915033	16:85,985,431	intergenic	T	C	5.055625	7.60 × 10^−7^	0.000396	
	rs139818184	17:37,514,738	intronic	C	A	−4.57152	7.76 × 10^−6^	0.00498	***FBXL20***
	rs7217237	17:38,827,062	intergenic	A	C	−4.5853	7.28 × 10^−6^	0.005523	*SMARCE1*, *KRT222*, *KRT24*
	rs55941736	19:48,831,886	intronic	A	G	−5.46051	5.58 × 10^−8^	0.000587	***EMP3***, *ODAD1*, *TMEM143*
	rs76473307	20:57,406,068	ncRNA_intronic	A	C	−5.32251	1.93 × 10^−7^	0.000483	*GNAS*
MZL	rs181136801	2:71,705,498	intronic	T	C	5.154366	3.05 × 10^−7^	0.003532	***DYSF***
	rs1202393	7:149,000,000	intronic	G	A	4.492213	7.80 × 10^−6^	0.009952	***ZNF282***, *ZNF398*
	rs1123007	11:71,185,357	intronic	G	A	4.510585	7.16 × 10^−6^	0.004986	***NADSYN1***, *DHCR7*
	rs80081219	11:71,524,587	ncRNA_exonic		T	4.462791	9.29 × 10^−6^	0.006537	*DEFB108B*, *NUMA1*, *RNF121*, *IL18BP*, *XNDC1N*
	rs575280	11:116,527,728	ncRNA_intronic	A	G	4.421853	1.12 × 10^−5^	0.006556	
	rs34893450	16:54,995,307	intergenic	G	A	4.967814	8.02 × 10^−7^	0.007275	

CLL: Chronic lymphocytic leukemia; CHR:BP: Chromosome:Base pair; condFDR: Conditional false discovery rate; DLBCL: Diffuse large B-cell lymphoma; FL: Follicular lymphoma; MZL: Marginal zone lymphoma; SNP: Single nucleotide polymorphism. For lead SNPs identified across more than one dataset, the one with the lowest condFDR is reported. § Positional gene mapping of non-HLA lead SNPs (with the gene containing the SNP shown in **bold**) that had a condFDR <0.01 but did not previously reach genome-wide significance (*p* < 5 × 10^−8^), as well as candidate SNPs in high linkage disequilibrium (LD; r^2^ > 0.8) with these lead SNPs.

## Data Availability

Data are available from the corresponding author upon reasonable request.

## Supplementary Materials

The following supporting information can be downloaded at: https://www.mdpi.com/article/10.3390/cancers18101536/s1. Figure S1. eQTLs and Hi-C chromatin interactions at previously reported DLBCL risk loci on chromosomes 2, 3, and 8 (arranged clockwise). The outermost layer is the Manhattan plot displaying the lead SNPs, with their respective SNP IDs labeled. The outer circle is the chromosome coordinate with genomic risk loci highlighted in blue. Genes mapped by either eQTLs (green), chromatin interaction (orange), or both (red) are shown on the inner circle. eQTL and chromatin interaction links are shown as links in green and orange, respectively. DLBCL: Diffuse large B-cell lymphoma; SNP: Single nucleotide polymorphism; eQTL: expression quantitative trait locus. Figure S2. eQTLs and Hi-C chromatin interactions at previously reported FL risk loci on chromosomes 3, 7, 8, 9, 11, and 18 (arranged clockwise). The outermost layer is the Manhattan plot displaying the lead SNPs, with their respective SNP IDs labeled. The outer circle is the chromosome coordinate with genomic risk loci highlighted in blue. Genes mapped by either eQTLs (green), chromatin interaction (orange), or both (red) are shown on the inner circle. eQTL and chromatin interaction links are shown as links in green and orange, respectively. FL: Follicular lymphoma; SNP: Single nucleotide polymorphism; eQTL: expression quantitative trait locus. Figure S3. eQTLs and Hi-C chromatin interactions at previously reported CLL risk loci. (a) Chromosomes 1-4. (b) Chromosomes 1, 2, 5, 6, 8, 9, 10-12, and 19. (c) Chromosomes 1-4, 8, 11, 14-19. (d) Chromosomes 16, 18, and 20 (arranged clockwise). The outermost layer is the Manhattan plot displaying the lead SNPs, with their respective SNP IDs labeled. The outer circle is the chromosome coordinate with genomic risk loci highlighted in blue. Genes mapped by either *cis*/*trans*-eQTLs (green), chromatin interaction (orange), or both (red) are shown on the inner circle. *Cis*/*trans*-eQTL and chromatin interaction links are shown as links in green and orange, respectively. CLL: chronic lymphocytic leukemia; SNP: single nucleotide polymorphism; eQTL: expression quantitative trait locus. Figure S4. Remaining significantly enriched Gene Ontology biological processes (false discovery rate < 0.05) among genes mapped in chronic lymphocytic leukemia. Figure S5. eQTLs and Hi-C chromatin interactions at previously reported MZL risk loci on chromosome 6. The outermost layer is the Manhattan plot displaying the lead SNPs with SNP ID labeled. The outer circle is the chromosome coordinate with genomic risk loci highlighted in blue. Genes mapped by either eQTLs (green), chromatin interaction (orange), or both (red) are shown on the inner circle. eQTL and chromatin interaction links are shown as links in green and orange, respectively. MZL: marginal zone lymphoma; SNP: single nucleotide polymorphism; eQTL: expression quantitative trait locus. Table S1. The number of SNPs excluded in each QC and data alignment step. Table S2. The number of single nucleotide polymorphisms for each disease pair included in the conditional false discovery rate analyses. Table S3. Parameter settings in FUMA SNP2GENE. Table S4. Novel and previously reported loci associated with diffuse large B-cell lymphoma, identified by analyses conditioned on dermatomyositis and polymyositis. Table S5. Novel and previously reported loci associated with follicular lymphoma, identified by analyses conditioned on dermatomyositis and polymyositis. Table S6. Novel and previously reported loci associated with chronic lymphocytic leukemia, identified by analyses conditioned on dermatomyositis and polymyositis. Table S7. Novel and previously reported loci associated with marginal zone lymphoma, identified by analyses conditioned on dermatomyositis and polymyositis. Table S8. Expression quantitative trait locus (FDR < 0.05) mapping of novel loci for diffuse large B-cell lymphoma. Table S9. Hi-C chromatin interaction mapping of (FDR < 1 × 10^−6^) of novel loci for diffuse large B-cell lymphoma. Table S10. Expression quantitative trait locus (FDR < 0.05) mapping of loci previously reported for diffuse large B-cell lymphoma. Table S11. Hi-C chromatin interaction mapping of (FDR < 1 × 10^−6^) of previously reported loci for diffuse large B-cell lymphoma. Table S12. Simplified Gene Ontology biological processes (FDR < 0.05) annotated to risk loci of diffuse large B-cell lymphoma. Table S13. Expression quantitative trait locus (FDR < 0.05) mapping of novel loci for follicular lymphoma. Table S14. Expression quantitative trait locus (FDR < 0.05) mapping of previously reported loci for follicular lymphoma. Table S15. Hi-C chromatin interaction mapping of (FDR < 1 × 10^−6^) of previously reported loci for follicular lymphoma. Table S16. Simplified Gene Ontology biological processes (FDR < 0.05) annotated to risk loci of follicular lymphoma. Table S17. Expression quantitative trait locus (FDR <0.05) mapping of novel loci for chronic lymphocytic leukemia. Table S18. Hi-C chromatin interaction mapping of (FDR < 1 × 10^−6^) of novel loci for chronic lymphocytic leukemia. Table S19. Expression quantitative trait locus (FDR < 0.05) mapping of previously reported loci for chronic lymphocytic leukemia. Table S20. Hi-C chromatin interaction mapping of (FDR < 1 × 10^−6^) of previously reported loci for chronic lymphocytic leukemia. Table S21. Simplified Gene Ontology biological processes (FDR < 0.05) annotated to risk loci of chronic lymphocytic leukemia. Table S22. Expression quantitative trait locus (FDR < 0.05) mapping of novel loci for marginal zone lymphoma. Table S23. Expression quantitative trait locus (FDR < 0.05) mapping of previously reported loci for marginal zone lymphoma. Table S24. Hi-C chromatin interaction mapping of (FDR < 1 × 10^−6^) of previously reported loci for marginal zone lymphoma.
