# Supplementary material for: Identification of Novel Risk Loci for Common B-Cell Lymphoma Subtypes Through Cross-Trait Analysis with Idiopathic Inflammatory Myopathies
Source: Cancers (Basel). 2026 May 9;18(10):1536. doi: 10.3390/cancers18101536 (PMC13204642; doi:10.3390/cancers18101536)
Supplement: Supplementary file 1 [file cancers-18-01536-s001.zip › Supplementary_IIMBLYMP_BLYMP_tables 1-7_figures 1-5_appendices.pdf]

Identification of novel risk loci for common B-cell lymphoma subtypes through cross-trait analysis with idiopathic inflammatory myopathies

Weng Ian Che[1], James N. Jarvis[2], International Lymphoma Epidemiology Consortium (InterLymph)#, IMACS Genetics Group (MYOGEN)#, Ingrid E. Lundberg[3, 4], Karin E. Smedby[5, 6], Janine A Lamb*[7], Marie Holmqvist*[4, 5]

[1]Department of Public Health and Medicinal Administration, Faculty of Health Sciences, University of Macau, Macau SAR, China. [2]Department of Pediatrics and Center for Indigenous Health, University of Washington School of Medicine, Seattle, WA, USA. [3]Division of rheumatology, Department of Medicine, Solna, Karolinska Institutet, Stockholm, Sweden. [4]ME Gastro, Derm and Rheuma, Theme Inflammation and Aging, Karolinska University Hospital, Stockholm, Sweden. [5]Clinical epidemiology division, Department of Medicine, Solna, Karolinska Institutet, Stockholm, Sweden. [6]Hematology Center, Karolinska University Hospital, Stockholm, Sweden. [7]Epidemiology and Public Health Group, School of Health Sciences, The University of Manchester, Manchester, UK.

*Dr. Janine A Lamb and Dr. Marie Holmqvist contributed equally to this work.

#Investigators and individual studies contributing to the present study, in addition to the authors, are presented in the supplementary appendices.

Supplementary materials

SUPPLEMENTARY TABLES

Table S1. The number of SNPs excluded in each QC and data alignment step[a]

		MYOGEN				InterLymph			
	**Number of SNPs/patients**	**GWAS DM**	**GWAS PM**	**Immunochip DM**	**Immunochip PM**	**DLBCL**	**FL**	**CLL**	**MZL**
**Cases**		402	255	870	923	3,587	2,847	3,100	825
**Controls**		4,336	4,336	7,486	7,486	7,666	8,107	7,667	6,221
	**Imputed SNPs**	**8,668,073**	**8,668,073**	**1,201,875**	**1,201,875**	**9,116,853**	**9,078,855**	**9,098,434**	**8,478,065**
**Stable biallelic SNPs**	Single-study group	-	-	-	-	498,560	460,723	487,417	-
	SNP duplicates	-	-	-	-	2	8	3	-
	Missing OR	2[b]	79[b]	-	-	-	-	-	-
	Multiallelic	573,612	573,686	79,739	79,739	-	-	-	-
**LiftOver to GRCh37**	Conversion unstable SNPs	1,878	1,878	48	48	-	-	-	-
	Failed to be converted	14,036	14,036	1,115	1,115	-	-	-	-
**QC and Alignment**	Missing on reference genome	50,963	50,945	38,455	38,455	49,253	49,005	48,787	48,121
	Ambiguous SNPs	1,241,828	1,241,813	164,677	164,677	1,324,362	1,324,362	1,323,363	1,302,798
	Zero effect value	-	-	-	-	-	-	-	3,764
	Not matched to reference genome	3,015	3,015	49	49	-	-	-	-
**Qced SNPs**	**After QC and alignment**	**6,782,631**	**6,782,590**	**917,536**	**917,536**	**7,244,776**	**7,244,757**	**7,238,864**	**7,123,382**

a SNP: Single nucleotide polymorphism; QC: Quality control; DM: Dermatomyositis; PM: Polymyositis; DLBCL: Diffuse large B-cell lymphoma; FL: Follicular lymphoma; CLL: Chronic lymphocytic leukaemia; MZL: Marginal zone lymphoma.

b SNPs with missing OR were multiallelic.

Table S2. The number of single nucleotide polymorphisms for each disease pair included in the conditional false discovery rate analyses

	Number of SNPs analysed	
	**GWAS**	**ImmunoChip**
**DLBCL-DM**	6260170	850547
**FL-DM**	6259969	850673
**CLL-DM**	6260163	850596
**MZL-DM**	6239939	849056
**DLBCL-PM**	6260153	850547
**FL-PM**	6259952	850673
**CLL-PM**	6260145	850596
**MZL-PM**	6239921	849056

GWAS: Genome-wide association analysis; DLBCL: Diffuse large B-cell lymphoma; FL: Follicular lymphoma; CLL: Chronic lymphocytic leukaemia; MZL: Marginal zone lymphoma; DM: Dermatomyositis; PM: Polymyositis.

Table S3. Parameter settings in FUMA SNP2GENE

**Parameters for candidate SNPs identification for all identified SNPs**	
r2	0.8
Reference panel	1KG/Phase3 EUR
Include variants in reference panel (non-GWAS tagged SNPs in linkage disequilibrium, LD)	Yes
Maximum distance between LD blocks to merge into a locus (< kb)	250
	Positional mapping
Maximum distance to genes (kb)	10
CADD, Regulome or 15-core chromatin state filtering	No
**eQTL mapping for newly identified SNPs**	
Tissue type	eQTLcatalogue/CEDAR_B-cell_CD19.txt.gz, eQTLcatalogue/Fairfax_2012_B-cell_CD19.txt.gz, eQTLcatalogue/GENCORD_ge_LCL.txt.gz, eQTLcatalogue/GEUVADIS_ge_LCL.txt.gz, eQTLcatalogue/Lepik_2017_ge_blood.txt.gz, eQTLcatalogue/TwinsUK_ge_blood.txt.gz, eQTLcatalogue/TwinsUK_ge_LCL.txt.gz, scRNA_eQTLs/B_cell.txt.gz, scRNA_eQTLs/PBMC.txt.gz, DICE/B_cell_naive.txt.gz, eQTLGen/eQTLGen_cis_eQTLs.txt.gz, eQTLGen/eQTLGen_trans_eQTLs.txt.gz, BloodeQTL/BloodeQTL.txt.gz, BIOSQTL/BIOS_eQTL_geneLevel.txt.gz, MuTHER/MuTHER_LCL.txt.gz, GTEx/v8/Cells_EBV-transformed_lymphocytes.txt.gz,

	GTEx/v8/Whole_Blood.txt.gz
P-value threshold	FDR < 0.05
CADD, Regulome or 15-core chromatin state filtering	No
**3D Chromatin interaction mapping for newly identified SNPs**	
Builtin chromatin interaction data	HiC/GSE87112/GM12878.txt.gz
P-value threshold	FDR < 1×10^{-6}
Promoter region window	250bp upstream and 500bp downstream from transcriptional start site
Annotate enhancer/promoter regions (Roadmap 111 epigenomes)	E031, E032, E035, E036, E050, E051
Filter SNPs by enhancers	Yes
Filter genes by promoters	Yes
CADD, Regulome or 15-core chromatin state filtering	No
**Gene types**	
Ensemble version	v102
Gene type	protein_coding
**Others**	
Major histocompatibility complex exclusion	Yes for novel SNPs in all mappings

Table S4. Novel and previously reported loci associated with diffuse large B-cell lymphoma, identified by analyses conditioned on dermatomyositis and polymyositis

Conditioning disease	Data source	SNP ID#	CHR:BP in hg19	Function	Reference allele	Effect allele	Z score	P-value	CondFDR	Mapped protein coding genes§
Dematomyositis	GWAS	rs80209550	2:20009773	intergenic	G	A	5,35	1,17E-07	0,008333	
		rs9831894	3:121800487	intronic	A	C	-5,86	6,16E-09	0,000398	***CD86***
		rs76106586	6:483593	intergenic	A	G	8,87	1,48E-18	1,32E-13	*EXOC2*
		rs2523593	6:31326703	intronic	T	C	5,29	1,54E-07	6,42E-05	
		rs13255292	8:129076573	intronic	C	T	6,25	5,95E-10	8,58E-05	
	ImmunoChip	**rs11582702**	1:160727986	intergenic	C	T	-4,57	6,09E-06	0,007251	*SLAMF7*
		rs79480871	2:24694472	intergenic	C	T	5,36	1,13E-07	0,001766	
		rs6795177	3:27800734	intergenic	A	G	5,11	4,28E-07	0,000821	
		rs9831894	3:121800487	intronic	A	C	-5,86	6,54E-09	6,13E-05	***CD86***
		rs76106586	6:483593	intergenic	A	G	8,87	1,69E-18	1,35E-15	*EXOC2*
		rs2523593	6:31326703	intronic	T	C	5,29	1,62E-07	9,13E-06	
		**rs11774454**	8:11270664	intronic	C	T	-4,87	1,41E-06	0,006425	*FAM167A-AS1*, *FAM167A*
		rs13255292	8:129076573	intronic	C	T	6,25	6,37E-10	8,90E-06	
Polymyositis	GWAS	rs6795177	3:27800734	intergenic	A	G	5,11	4,09E-07	0,005669	
		rs9831894	3:121800487	intronic	A	C	-5,86	6,17E-09	0,001327	***CD86***
		rs76106586	6:483593	intergenic	A	G	8,87	1,48E-18	2,61E-13	*EXOC2*
		**rs9885976**	6:16940672	intergenic	C	T	-4,59	5,50E-06	0,005573	
		rs2523593	6:31326703	intronic	T	C	5,29	1,54E-07	7,25E-05	
		rs13255292	8:129076573	intronic	C	T	6,25	5,96E-10	1,38E-05	
		**rs117408955**	13:112538767	intergenic	G	A	-4,32	1,85E-05	0,008798	
	ImmunoChip	rs79480871	2:24694472	intergenic	C	T	5,36	1,13E-07	0,002035	
		rs6795177	3:27800734	intergenic	A	G	5,11	4,28E-07	0,003993	
		rs9831894	3:121800487	intronic	A	C	-5,86	6,54E-09	5,25E-05	***CD86***
		rs76106586	6:483593	intergenic	A	G	8,87	1,69E-18	7,55E-15	*EXOC2*
		rs2523593	6:31326703	intronic	T	C	5,29	1,62E-07	7,94E-06	

**rs2251301**	8:11119037	ncRNA_intronic	C	T	-4,93	1,05E-06	0,009425	*FAM167A*
rs13255292	8:129076573	intronic	C	T	6,25	6,37E-10	1,05E-05	

SNP: Single nucleotide polymorphism; GWAS: Genome-wide association study; CHR:BP: Chromosome:Base pair; condFDR: Conditional false discovery rate
# Novel SNPs are shown in **bold**.
§ Positional gene mapping of non-HLA lead SNPs (with the gene containing the SNP shown in **bold**) that had a condFDR < 0.01 but did not previously reach genome-wide significance ($p < 5 \times 10^{-8}$), as well as candidate SNPs in high linkage disequilibrium (LD; $r^2 > 0.8$) with these lead SNPs.

Table S5. Novel and previously reported loci associated with follicular lymphoma, identified by analyses conditioned on dermatomyositis and polymyositis

Conditioning disease	Data source	SNP ID	CHR:BP in hg19	Function	Reference allele	Effect allele	Z score	P-value	CondFDR	Mapped protein coding genes§
Dematomyositis	GWAS	**rs10189015**[3-6]	2:111918472	intronic	G	A	5,256825	1,97E-07	0,005492	***BCL2L11***, *ACOXL*
		**rs2681416**[3,7]	3:121817613	intronic	G	A	5,425597	7,90E-08	0,001015	***CD86***
		rs12195582	6:32444544	intergenic	C	T	17,30566	9,45E-66	3,27E-26	
		rs10892296[7]	11:118718729	intergenic	C	T	7,19371	1,09E-12	1,94E-08	*DDX6*, *CXCR5*
		rs4245081	11:128489380	intronic	C	T	-5,64665	2,30E-08	0,002091	
		rs17749561[3,7]	18:60783211	intergenic	G	A	-5,27744	1,76E-07	0,002669	*BCL2*
	ImmunoChip	**rs10189015**	2:111918472	intronic	G	A	5,256825	2,31E-07	0,001103	***BCL2L11***, *ACOXL*
		rs2887944	3:27758275	UTR3	G	T	4,489306	9,99E-06	0,007108	***EOMES***
		**rs2681416**	3:121817613	intronic	G	A	5,425597	9,36E-08	0,000638	***CD86***
		rs12195582	6:32444544	intergenic	C	T	17,30566	5,03E-65	4,62E-27	
		rs13254990[3,7]	8:129076451	ncRNA_intronic	C	T	5,147045	4,09E-07	0,003002	
		rs10892296	11:118718729	intergenic	C	T	7,19371	1,46E-12	2,38E-10	*DDX6*, *CXCR5*
		rs4245081	11:128489380	intronic	C	T	-5,64665	2,76E-08	0,000135	
Polymyositis	GWAS	**rs10189015**	2:111918472	intronic	G	A	5,256825	1,97E-07	0,002346	***BCL2L11***, *ACOXL*
		**rs2681416**	3:121817613	intronic	G	A	5,425597	7,90E-08	0,005517	***CD86***
		rs12195582	6:32444544	intergenic	C	T	17,30566	9,45E-66	4,36E-28	
		rs13254990	8:129076451	ncRNA_intronic	C	T	5,147045	3,51E-07	0,006593	
		rs10892296	11:118718729	intergenic	C	T	7,19371	1,09E-12	3,58E-08	*DDX6*, *CXCR5*
		rs4245081	11:128489380	intronic	C	T	-5,64665	2,30E-08	0,000951	
	ImmunoChip	**rs10189015**	2:111918472	intronic	G	A	5,256825	2,31E-07	0,002893	***BCL2L11***, *ACOXL*
		**rs2681416**	3:121817613	intronic	G	A	5,425597	9,36E-08	0,001568	***CD86***
		rs12195582	6:32444544	intergenic	C	T	17,30566	5,03E-65	4,54E-29	
		rs13254990	8:129076451	ncRNA_intronic	C	T	5,147045	4,09E-07	0,005129	
		rs10892296	11:118718729	intergenic	C	T	7,19371	1,46E-12	9,45E-09	*DDX6*, *CXCR5*
		rs4245081	11:128489380	intronic	C	T	-5,64665	2,76E-08	0,00046	

SNP: Single nucleotide polymorphism; GWAS: Genome-wide association study; CHR:BP: Chromosome:Base pair; condFDR: Conditional false discovery rate
# Novel SNPs are shown in **bold**.
§ Positional gene mapping of non-HLA lead SNPs (with the gene containing the SNP shown in **bold**) that had a condFDR < 0.01 but did not previously reach genome-wide significance ($p < 5 \times 10^{-8}$), as well as candidate SNPs in high linkage disequilibrium (LD; $r^2 > 0.8$) with these lead SNPs.

Table S6. Novel and previously reported loci associated with chronic lymphocytic leukemia, identified by analyses conditioned on dermatomyositis and polymyositis

Conditioning disease	Data source	SNP ID	CHR:BP in hg19	Function	Reference allele	Effect allele	Z score	P-value	CondFDR	Mapped protein coding genes§
		rs3770745[5]	2:37596089	intronic	C	T	5,27021	1,58E-07	0,002562	***QPCT***
		rs58055674	2:111831793	intronic	T	C	9,159091	8,16E-20	1,80E-15	***ACOXL***
		rs13015798	2:201909515	intronic	A	G	-5,55998	3,19E-08	0,000586	***HYCC2***, *ALS2CR12*, *CASP8*, *CASP10*
		rs7557418	2:231098071	intronic	C	A	9,883068	8,27E-23	6,72E-18	***SP140***, *SP110*
		rs115271170[8]	2:242347614	intronic	C	T	5,384482	8,50E-08	0,000697	***FARP2***, *SEPTIN2*
		rs4680838[3,6]	3:27783476	intergenic	A	G	4,9495	8,50E-07	0,009494	*EOMES*
		**rs1274963**[6]	3:39191029	intronic	A	G	-4,8292	1,56E-06	0,006201	*CSRNP1*
		rs9815073[6]	3:188115682	intronic	C	A	-5,01326	6,13E-07	0,003924	***LPP***
		rs2003869[5]	4:109026414	intronic	A	G	5,262822	1,65E-07	0,001003	***LEF1***
		**rs2242652**[9]	5:1280028	intronic	G	A	5,323386	1,19E-07	0,00126	*TERT*
		rs9391997	6:409119	UTR3	A	G	9,590553	1,43E-21	7,13E-17	***IRF4***
		rs73718779[6]	6:2969278	intronic	C	T	4,984314	7,12E-07	0,006684	***SERPINB6***
Dermatomyositis	GWAS	rs9273482	6:32628127	intronic	T	C	6,927801	5,52E-12	2,91E-09	
		rs210143[10,11]	6:33546930	intronic	T	C	5,335735	1,11E-07	0,001218	
		rs2511714[5,12]	8:103578874	intergenic	T	G	5,18533	2,49E-07	0,002062	*ODF1*
		rs113952129[8]	8:128195334	intronic	T	C	7,019125	2,90E-12	2,37E-08	
		rs10738616	9:22287608	intronic	G	A	-5,69535	1,47E-08	0,000147	
		rs142239370	10:72382869	intergenic	A	C	4,874788	1,24E-06	0,008328	*PRF1*
		rs4406737	10:90759724	intronic	A	G	7,453903	1,22E-13	2,40E-09	***FAS***, *ACTA2*
		rs2521269	11:2321095	intronic	C	A	-5,51326	4,15E-08	0,000644	***C11orf21***, *TSPAN32*
		rs35923643	11:123355391	intronic	A	G	13,30922	5,20E-40	9,01E-26	***GRAMD1B***
		rs539846	15:40397936	intronic	G	T	6,174993	8,12E-10	2,10E-05	***BMF***
		rs72742684	15:56780767	intergenic	C	T	7,376355	2,17E-13	8,75E-09	*RFX7*, *TEX9*, *MNS1*
		rs2052702	15:69989505	intergenic	A	C	-8,66821	6,54E-18	2,64E-13	
		**rs67063178**	16:82893008	UTR3	C	T	-4,89011	1,15E-06	0,00518	***CDH13***

	rs398114	16:85929284	intergenic	A	C	-9,23814	3,94E-20	7,69E-16	*IRF8*
	rs4368253[5]	18:57622287	intergenic	T	C	4,937814	9,02E-07	0,006051	
	rs4987856[5]	18:60793494	UTR3	C	T	-6,62188	4,48E-11	3,76E-07	***BCL2***
	**rs55941736**	19:48831886	intronic	A	G	-5,46051	5,58E-08	0,000587	***EMP3***, *ODAD1*, *TMEM143*
	**rs76473307**	20:57406068	ncRNA_intronic	A	C	-5,32251	1,19E-07	0,001373	*GNAS*
	**rs12021613**	1:20961950	intronic	C	T	-4,86479	1,95E-06	0,003199	***PINK1***, *CDA*, *DDOST*
	rs34517439[3]	1:78450517	intronic	C	A	4,803141	2,62E-06	0,004486	***DNAJB4***, *MIGA1*, *NEXN*, *FUBP1*, *GIPC2*
	rs58055674	2:111831793	intronic	T	C	9,159091	3,27E-19	4,40E-15	***ACOXL***
	rs8179786[5]	2:202143032	intronic	C	T	-4,68669	4,55E-06	0,004344	***CASP8***, *FLACC*, *EOMES*
	rs7557418	2:231098071	intronic	C	A	9,883068	4,15E-22	6,36E-18	***SP140***, *SP110*
	**rs17862882**	2:234682496	downstream	C	A	4,358045	2,02E-05	0,008728	*UGT1A1*, *UGT1A3*, *UGT1A4*, *UGT1A5*, *UGT1A6*, *UGT1A7*, *UGT1A8*, *UGT1A9*, *UGT1A10*, *MROH2A*
	rs115271170	2:242347614	intronic	C	T	5,384482	1,39E-07	0,000318	***FARP2***, *SEPTIN2*
	rs4680838	3:27783476	intergenic	A	G	4,9495	1,29E-06	0,000442	*EOMES*
	rs9815073	3:188115682	intronic	C	A	-5,01326	9,40E-07	0,002066	***LPP***
ImmunoChip	rs7690934[5]	4:109025865	intronic	T	C	5,13672	5,05E-07	0,001182	***LEF1***
	**rs2242652**	5:1280028	intronic	G	A	5,323386	1,92E-07	0,000275	***TERT***
	**rs56703405**	5:78186110	intronic	T	G	4,626346	6,03E-06	0,006574	***ARSB***
	rs9391997	6:409119	UTR3	A	G	9,590553	6,52E-21	1,51E-17	***IRF4***
	**rs10946876**	6:26896889	intronic	G	A	4,912766	1,54E-06	0,00312	
	rs9273482	6:32628127	intronic	T	C	6,927801	1,23E-11	4,34E-10	
	rs210143	6:33546930	intronic	T	C	5,335735	1,80E-07	7,39E-06	
	rs113952129	8:128195334	intronic	T	C	7,019125	6,61E-12	1,22E-08	
	rs4406737	10:90759724	intronic	A	G	7,453903	3,07E-13	1,24E-09	***FAS***, *ACTA2*
	rs11187157[3]	10:94502244	intergenic	T	C	4,611642	6,45E-06	0,00939	
	rs2521269	11:2321095	intronic	C	A	-5,51326	6,93E-08	2,88E-05	***C11orf21***, *TSPAN32*
	rs11605119[13]	11:113542109	intergenic	G	A	4,203931	3,92E-05	0,009361	*TMPRSS5*, *ZW10*

		rs35923643	11:123355391	intronic	A	G	13,30922	9,58E-39	1,18E-26	***GRAMD1B***
		rs12591150	15:56499482	intronic	G	T	6,711105	5,22E-11	2,92E-07	*RFX7*, *TEX9*, *MNS1*
		rs2052702	15:69989505	intergenic	A	C	-8,66821	2,27E-17	2,02E-13	
		**rs1915033**[12,14]	16:85985431	intergenic	T	C	5,055625	7,60E-07	0,000396	
		**rs139818184**	17:37514738	intronic	C	A	-4,57152	7,76E-06	0,00498	***FBXL20***
		**rs7217237**	17:38827062	intergenic	A	C	-4,5853	7,28E-06	0,005523	*SMARCE1*, *KRT222*, *KRT24*
		**rs76473307**	20:57406068	ncRNA_intronic	A	C	-5,32251	1,93E-07	0,000514	*GNAS*
		rs3770745	2:37596089	intronic	C	T	5,27021	1,58E-07	0,002881	***QPCT***
		rs58055674	2:111831793	intronic	T	C	9,159091	8,16E-20	8,98E-15	***ACOXL***
		rs13015798	2:201909515	intronic	A	G	-5,55998	3,19E-08	0,000493	***HYCC2***, *ALS2CR12*, *CASP8*, *CASP10*
		rs7557418	2:231098071	intronic	C	A	9,883068	8,27E-23	1,23E-17	***SP140***, *SP110*
		rs115271170	2:242347614	intronic	C	T	5,384482	8,50E-08	0,001683	***FARP2***, *SEPTIN2*
		rs4680838	3:27783476	intergenic	A	G	4,9495	8,50E-07	0,008564	*EOMES*
		rs9815073	3:188115682	intronic	C	A	-5,01326	6,13E-07	0,00868	***LPP***
		rs2003869	4:109026414	intronic	A	G	5,262822	1,65E-07	0,002838	***LEF1***
		**rs2242652**	5:1280028	intronic	G	A	5,323386	1,19E-07	0,002359	***TERT***
Polymyositis	GWAS	rs9391997	6:409119	UTR3	A	G	9,590553	1,43E-21	1,28E-16	***IRF4***
		rs73718779	6:2969278	intronic	C	T	4,984314	7,12E-07	0,009137	***SERPINB6***
		**rs75320317**	6:7202278	intronic	T	C	-4,49139	7,90E-06	0,008553	***RREB1***
		rs9273482	6:32628127	intronic	T	C	6,927801	5,52E-12	1,39E-07	
		rs210143	6:33546930	intronic	T	C	5,335735	1,11E-07	0,002213	
		rs2511714	8:103578874	intergenic	T	G	5,18533	2,49E-07	0,00428	*ODF1*
		rs113952129	8:128195334	intronic	T	C	7,019125	2,90E-12	5,14E-08	
		rs10738616	9:22287608	intronic	G	A	-5,69535	1,47E-08	0,00035	
		rs4406737	10:90759724	intronic	A	G	7,453903	1,22E-13	9,23E-09	***FAS***, *ACTA2*
		rs2521269	11:2321095	intronic	C	A	-5,51326	4,15E-08	0,000845	***C11orf21***, *TSPAN32*
		rs35923643	11:123355391	intronic	A	G	13,30922	5,20E-40	1,17E-25	***GRAMD1B***

	rs539846	15:40397936	intronic	G	T	6,174993	8,12E-10	2,25E-05	***BMF***
	rs72742684	15:56780767	intergenic	C	T	7,376355	2,17E-13	1,12E-08	*RFX7*, *TEX9*, *MNS1*
	rs2052702	15:69989505	intergenic	A	C	-8,66821	6,54E-18	6,71E-13	
	rs398114	16:85929284	intergenic	A	C	-9,23814	3,94E-20	2,72E-15	*IRF8*
	rs4368253	18:57622287	intergenic	T	C	4,937814	9,02E-07	0,008912	
	rs4987856	18:60793494	UTR3	C	T	-6,62188	4,48E-11	1,93E-06	***BCL2***
	**rs55941736**	19:48831886	intronic	A	G	-5,46051	5,58E-08	0,001083	***EMP3***, *ODAD1*, *TMEM143*
	**rs76473307**	20:57406068	ncRNA_intronic	A	C	-5,32251	1,19E-07	0,001804	*GNAS*
ImmunoChip	**rs12021613**	1:20961950	intronic	C	T	-4,86479	1,95E-06	0,002663	***PINK1***, *CDA*, *DDOST*
	rs34517439	1:78450517	intronic	C	A	4,803141	2,62E-06	0,005262	***DNAJB4***, *MIGA1*, *NEXN*, *FUBP1*, *GIPC2*
	rs58055674	2:111831793	intronic	T	C	9,159091	3,27E-19	1,91E-15	***ACOXL***
	rs8179786	2:202143032	intronic	C	T	-4,68669	4,55E-06	0,007648	***CASP8***, *FLACC*, *EOMES*
	rs7557418	2:231098071	intronic	C	A	9,883068	4,15E-22	2,32E-18	***SP140***, *SP110*
	rs115271170	2:242347614	intronic	C	T	5,384482	1,39E-07	0,000384	***FARP2***, *SEPTIN2*
	rs4680838	3:27783476	intergenic	A	G	4,9495	1,29E-06	0,002827	*EOMES*
	rs9815073	3:188115682	intronic	C	A	-5,01326	9,40E-07	0,001849	***LPP***
	rs7690934	4:109025865	intronic	T	C	5,13672	5,05E-07	0,000292	***LEF1***
	**rs2242652**	5:1280028	intronic	G	A	5,323386	1,92E-07	0,000505	***TERT***
	**rs56703405**	5:78186110	intronic	T	G	4,626346	6,03E-06	0,009718	***ARSB***

rs9391997	6:409119	UTR3	A	G	9,590553	6,52E-21	3,01E-17	***IRF4***
**rs7451690**	6:7109433	intronic	C	T	-4,13257	5,29E-05	0,005872	***RREB1***
**rs10946876**	6:26896889	intronic	G	A	4,912766	1,54E-06	0,001625	
rs9273482	6:32628127	intronic	T	C	6,927801	1,23E-11	4,06E-10	
rs210143	6:33546930	intronic	T	C	5,335735	1,80E-07	0,000247	
rs113952129	8:128195334	intronic	T	C	7,019125	6,61E-12	2,59E-08	
rs4406737	10:90759724	intronic	A	G	7,453903	3,07E-13	1,63E-09	***FAS***, *ACTA2*
rs11187157	10:94502244	intergenic	T	C	4,611642	6,45E-06	0,008052	
rs2521269	11:2321095	intronic	C	A	-5,51326	6,93E-08	0,000164	***C11orf21***, *TSPAN32*
rs35923643	11:12335539 1	intronic	A	G	13,30922	9,58E-39	1,04E-26	***GRAMD1B***
rs12591150	15:56499482	intronic	G	T	6,711105	5,22E-11	8,77E-08	*RFX7*, *TEX9*, *MNS1*
rs2052702	15:69989505	intergenic	A	C	-8,66821	2,27E-17	1,72E-13	
**rs1915033**	16:85985431	intergenic	T	C	5,055625	7,60E-07	0,001766	
**rs76473307**	20:57406068	ncRNA_int ronic	A	C	-5,32251	1,93E-07	0,000483	*GNAS*

SNP: Single nucleotide polymorphism; GWAS: Genome-wide association study; CHR:BP: Chromosome:Base pair; condFDR: Conditional false discovery rate

# Novel SNPs are shown in **bold**.

§ Positional gene mapping of non-HLA lead SNPs (with the gene containing the SNP shown in **bold**) that had a condFDR < 0.01 but did not previously reach genome-wide significance ($p < 5 \times 10^{-8}$), as well as candidate SNPs in high linkage disequilibrium (LD; $r^2 > 0.8$) with these lead SNPs.

Table S7. Novel and previously reported loci associated with marginal zone lymphoma, identified by analyses conditioned on dermatomyositis and polymyositis

Conditioning disease	Data source	SNP ID	CHR:BP in hg19	Function	Reference allele	Effect allele	Z score	P-value	CondFDR	Mapped protein coding genes§
Dermatomyositis	GWAS	rs2523593[15]	6:31326703	intronic	T	C	5,417527	7,39E-08	2,98E-05	
		rs190777845	6:36394999	intronic	T	G	5,600593	2,65E-08	0,003791	***PXT1***, *STK38*, *KCTD20*
		rs56195322	10:75000196	intronic	C	T	5,528631	3,98E-08	0,00773	***FAM149B1***, *MRPS16*, *DNAJC9*
		**rs575280**	11:116527728	ncRNA_intronic	A	G	4,421853	1,12E-05	0,006556	
	ImmunoChip	rs2523593	6:31326703	intronic	T	C	5,417527	6,99E-08	3,54E-06	
		**rs1202393**	7:149000000	intronic	G	A	4,492213	7,80E-06	0,009952	***ZNF282***, *ZNF398*
		**rs1123007**	11:71185357	intronic	G	A	4,510585	7,16E-06	0,004986	***NADSYN1***, *DHCR7*
Polymyositis	GWAS	**rs181136801**	2:71705498	intronic	T	C	5,154366	3,05E-07	0,003532	***DYSF***
		rs2523593	6:31326703	intronic	T	C	5,417527	7,39E-08	3,46E-05	
		rs190777845	6:36394999	intronic	T	G	5,600593	2,65E-08	0,005441	***PXT1***, *STK38*, *KCTD20*
		rs56195322	10:75000196	intronic	C	T	5,528631	3,98E-08	0,001301	***FAM149B1***, *MRPS16*, *DNAJC9*
		**rs80081219**	11:71524587	ncRNA_exonic		T	4,462791	9,29E-06	0,006537	*DEFB108B*, *NUMA1*, *RNF121*, *IL18BP*, *XNDC1N*
		**rs34893450**	16:54995307	intergenic	G	A	4,967814	8,02E-07	0,007275	
	ImmunoChip	rs2523593	6:31326703	intronic	T	C	5,417527	6,99E-08	3,13E-06	
		**rs1123007**	11:71185357	intronic	G	A	4,510585	7,16E-06	0,009619	***NADSYN1***, *DHCR7*

SNP: Single nucleotide polymorphism; GWAS: Genome-wide association study; CHR:BP: Chromosome:Base pair; condFDR: Conditional false discovery rate
# Novel SNPs are shown in **bold**.
§ Positional gene mapping of non-HLA lead SNPs (with the gene containing the SNP shown in **bold**) that had a condFDR < 0.01 but did not previously reach genome-wide significance ($p < 5 \times 10^{-8}$), as well as candidate SNPs in high linkage disequilibrium (LD; $r^2 > 0.8$) with these lead SNPs.

SUPPLEMENTARY FIGURES

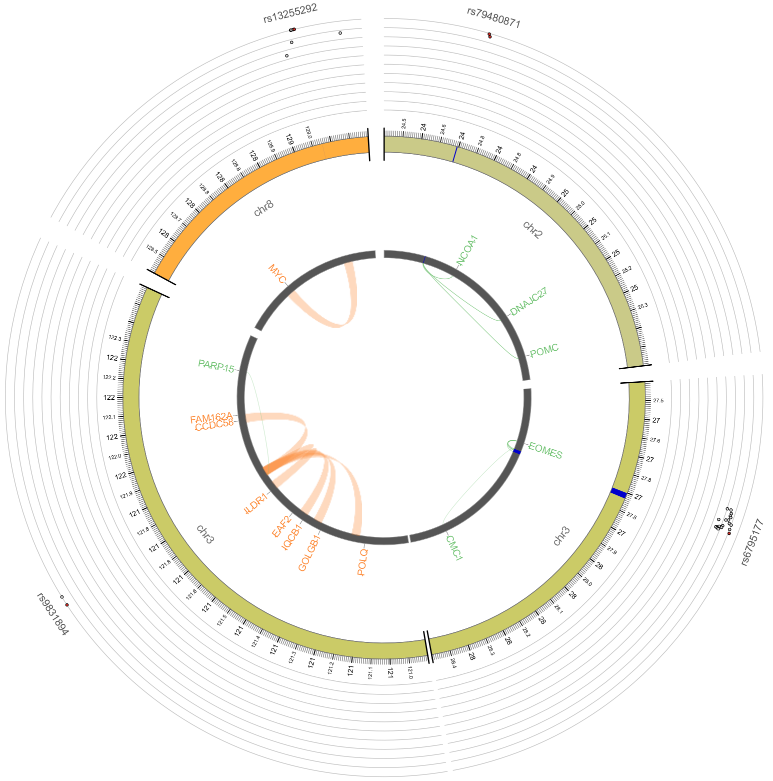

Figure S1. eQTLs and Hi-C chromatin interactions at previously reported DLBCL risk loci on chromosomes 2, 3, and 8 (arranged clockwise). The outermost layer is the Manhattan plot displaying the lead SNPs with SNP ID labelled. The outer circle is the chromosome coordinate with genomic risk loci highlighted in blue. Genes mapped by either eQTLs (green), chromatin interaction (orange) or both (red) are shown on the inner circle. eQTL and chromatin interaction links are shown as links in green and orange, respectively. DLBCL: Diffuse large B-cell lymphoma; SNP: Single nucleotide polymorphism; eQTL: expression quantitative trait locus.

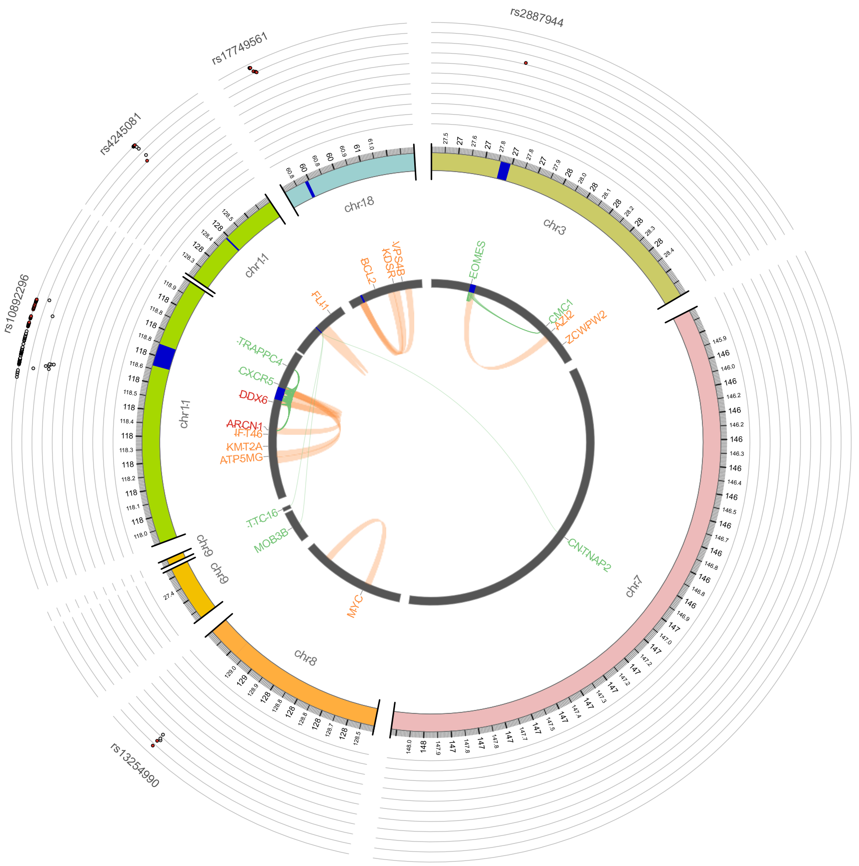

Figure S2. eQTLs and Hi-C chromatin interactions at previously reported FL risk loci on chromosomes 3, 7, 8, 9, 11, and 18 (arranged clockwise). The outermost layer is the Manhattan plot displaying the lead SNPs with SNP ID labelled. The outer circle is the chromosome coordinate with genomic risk loci highlighted in blue. Genes mapped by either eQTLs (green), chromatin interaction (orange) or both (red) are shown on the inner circle. eQTL and chromatin interaction links are shown as links in green and orange, respectively. FL: Follicular lymphoma; SNP: Single nucleotide polymorphism; eQTL: expression quantitative trait locus.

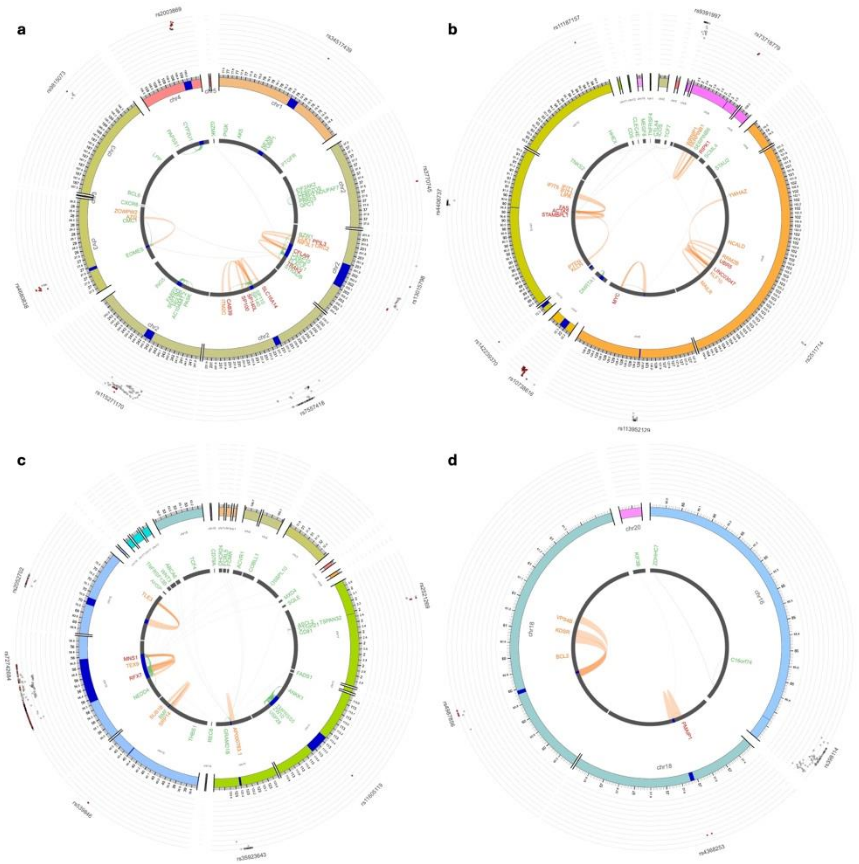

Figure S3. eQTLs and Hi-C chromatin interactions at previously reported CLL risk loci. a) Chromosomes 1-4. b) Chromosomes 1,2, 5, 6, 8, 9, 10-12, and 19. c) Chromosomes 1-4, 8, 11, 14-19. d) Chromosomes 16, 18, and 20 (arranged clockwise). The outermost layer is the Manhattan plot displaying the lead SNPs with SNP ID labelled. The outer circle is the chromosome coordinate with genomic risk loci highlighted in blue. Genes mapped by either *cis*/*trans*-eQTLs (green), chromatin interaction (orange) or both (red) are shown on the inner circle. *cis*/*trans*-eQTL and chromatin interaction links are shown as links in green and orange, respectively. CLL: Chronic lymphocytic leukemia; SNP: Single nucleotide polymorphism; eQTL: expression quantitative trait locus.

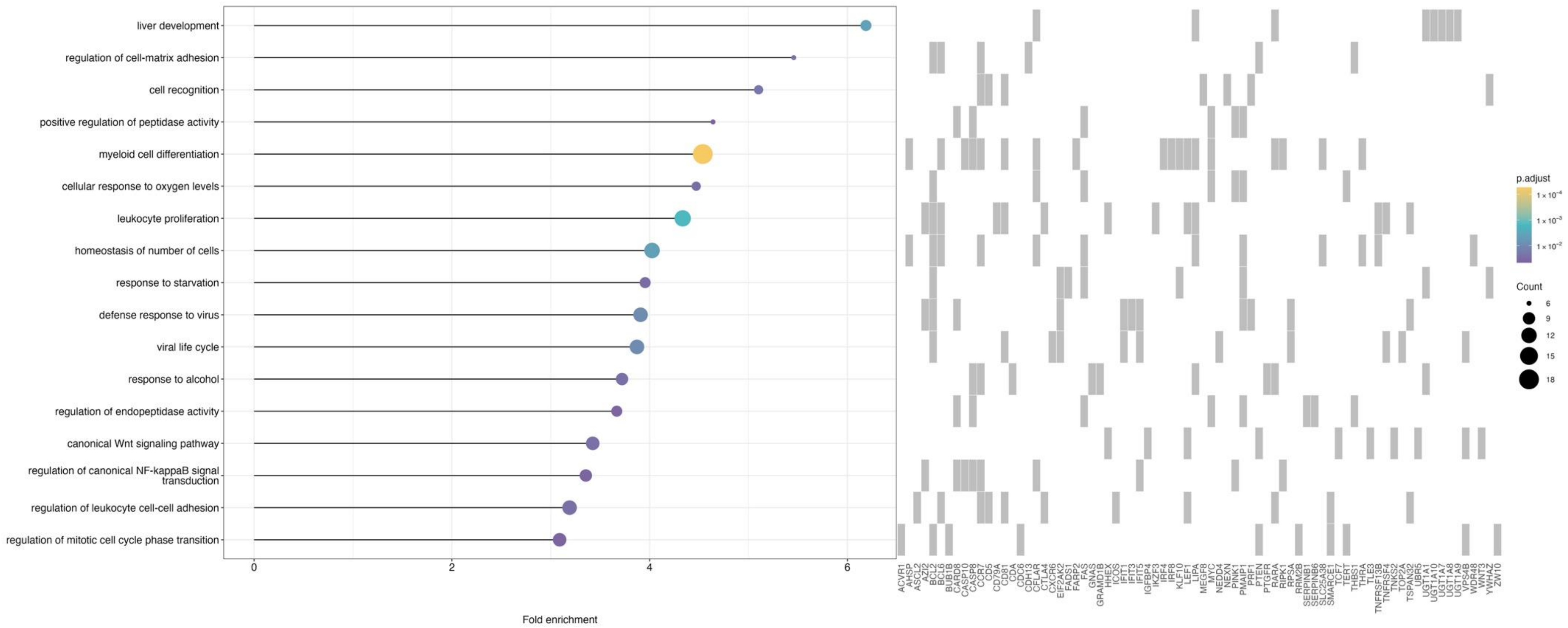

Figure S4. Remaining significantly enriched Gene Ontology biological processes (false discovery rate < 0.05) among genes mapped in chronic lymphocytic leukemia.

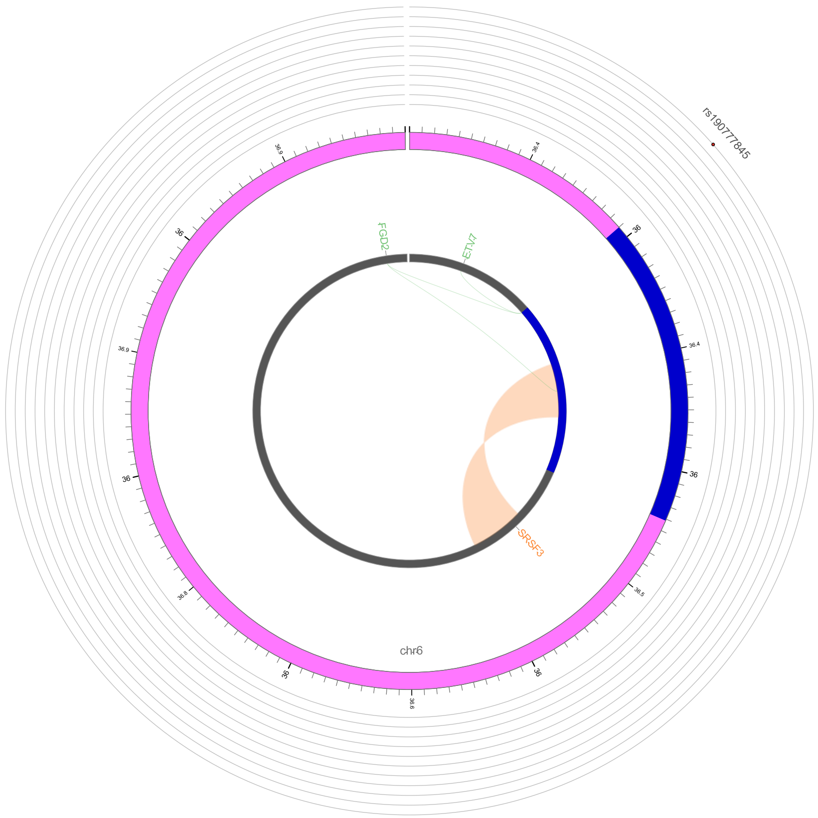

Figure S5. eQTLs and Hi-C chromatin interactions at previously reported MZL risk loci on chromosome 6. The outermost layer is the Manhattan plot displaying the lead SNPs with SNP ID labelled. The outer circle is the chromosome coordinate with genomic risk loci highlighted in blue. Genes mapped by either eQTLs (green), chromatin interaction (orange) or both (red) are shown on the inner circle. eQTL and chromatin interaction links are shown as links in green and orange, respectively. MZL: Marginal zone lymphoma; SNP: Single nucleotide polymorphism; eQTL: expression quantitative trait locus.

SUPPLEMENTARY APPENDIX I

Investigators from InterLymph Consortium contributing to the genetics studies of B-cell lymphomas

Sonja Berndt. Division of Cancer Epidemiology and Genetics, National Cancer Institute, Rockville, MD, USA.

Parveen Bhatti. School of Population and Public Health, University of British Columbia, Vancouver, BC, V6T 1Z3, Canada. Population Health Sciences, BC Cancer Research Institute, Vancouver, BC, V5Z 1L3, Canada.

Lauren Teras. Department of Population Science, American Cancer Society, Atlanta, GA, United States.

Alain Monnereau. Bordeaux Population Health Research Center, EPICENE, UMR 1219, Univ. Bordeaux, Inserm, Bordeaux, France; Registre des Hémopathies Malignes De La Gironde - Institut Bergonié, Bordeaux, France.

Roel Vermeulen. Institute for Risk Assessment Sciences, Division of Environmental Epidemiology and Julius Center for Health Sciences and Primary Care, University Medical Center Utrecht, Utrecht University, Utrecht, Netherlands.

Delphine Casabonne. Spanish Consortium for Research on Epidemiology and Public Health (CIBERESP), Madrid, Spain; Unit of Molecular and Genetic Epidemiology in Infections and Cancer (UNIC-Molecular), Cancer Epidemiology Research Programme, IDIBELL, Catalan Institute of Oncology, Becelona, Spain.

Alexandra Nieters. Institute for Immunodeficiency, Medical Centre and Faculty of Medicine, Albert-Ludwigs-University, Freiburg, Germany.

James McKay. International Agency for Research on Cancer, World Health Organization, Lyon, France.

Brenda M. Birmann. Channing Division of Network Medicine, Department of Medicine, Brigham and Women's Hospital and Harvard Medical School, Boston, MA, USA.

Pierluigi Cocco. Department of Medical Sciences and Public Health, University of Cagliari, Cagliari, Italy.

James Cerhan. Department of Quantitative Health Sciences, Mayo Clinic, Rochester, MN, USA.

Brian Link. Division of Hematology, Oncology and Bone Marrow Transplantation, University of Iowa, Iowa City, IA, USA.

Roger Milne. Cancer Council Victoria, East Melbourne, Victoria, Australia; Centre for Epidemiology and Biostatistics, Melbourne School of Population and Global Health, The University of Melbourne, Victoria, Australia; Precision Medicine, School of Clinical Sciences at Monash Health, Monash University, Clayton, Victoria, Australia.

Harindra Jayasekara. Cancer Epidemiology Division, Cancer Council Victoria, Melbourne, Victoria, Australia.Centre for Epidemiology and Biostatistics, Melbourne School of Population and Global Health, The University of Melbourne, Melbourne, Victoria, Australia. School of Public Health and Preventive Medicine, Faculty of Medicine, Nursing and Health Sciences, Monash University, Clayton, Victoria, Australia.

Vijai Joseph. Memorial Sloan-Kettering Cancer Center, New York, NY, USA.

Kenneth Offit. Memorial Sloan-Kettering Cancer Center, New York, NY, USA.

Nathaniel Rothman. Division of Cancer Epidemiology and Genetics, National Cancer Institute, NIH, DHHS, Rockville, MD, USA.

Claire Vajdic. Kirby Institute, UNSW Sydney, Sydney, Australia.

Alan Arslan. Departments of Obstetrics and Gynecology and Population Health, New York University Grossman School of Medicine, New York, NY, USA.

Mark Purdue. Division of Cancer Epidemiology and Genetics, National Cancer Institute, NIH, DHHS, Rockville, MD, USA.

Henrik Hjalgrim. Haematology, Danish Cancer Institute, Copenhagen, Denmark. Department of Haematology, Rigshospitalet, University of Copenhagen, Copenhagen, Denmark. Department of Clinical Medicine, University of Copenhagen, Copenhagen, Denmark.

Karin Ekstrom Smedby. Clinical epidemiology division, Department of Medicine, Solna, Karolinska Institutet, Stockholm, Sweden. Hematology Center, Karolinska University Hospital, Stockholm, Sweden.

Paige Bracci. Department of Epidemiology and Biostatistics, University of California, San Francisco, San Francisco, California, USA.

Eleanor Kane. Epidemiology and Cancer Statistics Group, Department of Health Sciences, University of York, York, UK.

Lesley Tinker. Division of Public Health Sciences, Women's Health Initiative Clinical Coordinating Center, Fred Hutchinson Cancer Center, Seattle, WA, USA.

Qing Lan. Division of Cancer Epidemiology and Genetics, National Cancer Institute, NIH, DHHS, Rockville, MD, USA.

Susan Slager. Department of Quantitative Health Sciences, Mayo Clinic, Rochester, Minnesota, USA. Division of Hematology, Mayo Clinic, Rochester, Minnesota, USA.

Immaculata De Vivo. Channing Division of Network Medicine, Department of Medicine, Brigham and Women's Hospital and Harvard Medical School, Boston, MA, USA; Department of Epidemiology, Harvard T.H. Chan School of Public Health, Boston, MA, USA.

Gilles Salles. Department of Medicine, Lymphoma Service, Memorial Sloan Kettering Cancer Center, New York, NY, USA. Department of Medicine, Weill Cornell Medical College, New York, NY, USA.

Nicola Camp. Huntsman Cancer Institute, Salt Lake City, UT 84108, USA; Department of Internal Medicine, University of Utah School of Medicine, Salt Lake City, UT 84108, USA.

SUPPLEMENTARY APPENDIX II

Investigators from IMACS Myositis Genetics Scientific Interest Group (MYOGEN) contributing to MYOGEN GWAS and ImmunoChip studies

Chris Amos, Catherine Zhu. Baylor College of Medicine, Houston, Texas.

Olivier Benveniste. Department of Internal Medicine and Clinical Immunology, Pitié-Salpêtrière Hospital, Paris, France.

Angela Chun. Department of Pediatrics, Division of Rheumatology. Ann & Robert H. Lurie Children's Hospital of Chicago, Northwestern University Feinberg School of Medicine, Chicago, Illinois.

Hector Chinoy. National Institute for Health Research Manchester Biomedical Research Centre, Manchester University NHS Foundation Trust, The University of Manchester, Manchester, UK, and Department of Rheumatology, Salford Royal Hospital, Northern Care Alliance NHS Foundation Trust, Manchester Academic Health Science Centre, Salford, UK, and Centre for Musculoskeletal Research, Faculty of Biology, Medicine and Health, The University of Manchester, Manchester, UK.

Jan L De Bleecker, Boel De Paepe. Department of Neurology, Ghent University, Ghent, Belgium.

Andrea Doria. Rheumatology Unit, Department of Medicine, University of Padova, Padova, Italy.

Zoltan Geiger. University of Debrecen, Faculty of Medicine, Department of Internal Medicine, Division of Clinical Immunology, Debrecen, Hungary.

Peter Gregersen. The Robert S. Boas Center for Genomics and Human Genetics, The Feinstein Institute, Manhasset, New York.

Michael G. Hanna. Department of Neuromuscular Diseases, UCL Queen Square Institute of Neurology, and Centre for Rheumatology, UCL Division of Medicine, University College London, London, UK.

Janine A Lamb. Epidemiology and Public Health Group, School of Health Sciences, University of Manchester, Manchester, UK.

Vidya Limaye. Rheumatology Unit, Royal Adelaide Hospital and Discipline of Medicine, Adelaide University, Adelaide, Australia.

Ingrid Lundberg, Leonid Padyukov: Division of Rheumatology, Department of Medicine, Solna, Karolinska Institutet, Karolinska University Hospital, Stockholm, Sweden.

Pedro Machado. Department of Neuromuscular Diseases, UCL Queen Square Institute of Neurology, and Centre for Rheumatology, UCL Division of Medicine, University College London, London, UK.

Pernille Mathiesen. Paediatric Department, Slagelse Hospital and Paediatric Rheumatology Unit, Rigshospitalet, Copenhagen, Denmark.

Britta Maurer. Department of Rheumatology and Immunology, University Hospital, Bern, Switzerland.

Frederick W. Miller. Environmental Autoimmunity Group, National Institute of Environmental Health Sciences, NIH, Bethesda, Maryland.

Øyvind Molberg. Department of Rheumatology, Oslo University Hospital, Oslo, Norway.

Lauren Pachman. Ann & Robert H. Lurie Children's Hospital of Chicago, Northwestern University Feinberg School of Medicine, Chicago, Illinois.

Timothy Radstake. Department of Rheumatology and Clinical Immunology, University Medical Center, Utrecht, the Netherlands.

Ann M Reed. Department of Pediatrics, Duke University, Durham, North Carolina.

Lisa G. Rider. Environmental Autoimmunity Group, National Institute of Environmental Health Sciences, NIH, Bethesda, Maryland.

Simon Rothwell: Centre for Genetics and Genomics Versus Arthritis, Centre for Musculoskeletal Research, Faculty of Biology, Medicine and Health, University of Manchester, Manchester, UK.

Albert Selva-O'Callaghan. Internal Medicine Department, Vall d'Hebron General Hospital, Universitat Autonoma de Barcelona, Barcelona, Spain.

Jiri Vencovksy. Institute of Rheumatology and Department of Rheumatology, First Medical Faculty, Charles University, Prague, Czech Republic.

Lucy R Wedderburn. NIHR Biomedical Research Centre at Great Ormond Street Hospital, UCL Great Ormond Street Institute of Child Health, University College London, London, UK.

SUPPLEMENTARY APPENDIX III

Support for individual studies from the InterLymph Consortium

ATBC – The ATBC Study is supported by the Intramural Research Program of the U.S. National Cancer Institute, National Institutes of Health, Department of Health and Human Services.

BC – Canadian Institutes for Health Research (CIHR); Canadian Cancer Society; Michael Smith Foundation for Health Research.

CPS-II - The Cancer Prevention Study-II (CPS-II) Nutrition Cohort is supported by the American Cancer Society. Genotyping for all CPS-II samples were supported by the Intramural Research Program of the National Institutes of Health, NCI, Division of Cancer Epidemiology and Genetics. The authors would also like to acknowledge the contribution to this study from central cancer registries supported through the Centers for Disease Control and Prevention National Program of Cancer Registries, and cancer registries supported by the National Cancer Institute Surveillance Epidemiology and End Results program.

ELCCS – Blood Cancer UK, United Kingdom.

ENGELA – Association pour la Recherche contre le Cancer (ARC), Institut National du Cancer (INCa), Fondation de France, Fondation contre la Leucémie, Agence nationale de sécurité sanitaire de l'alimentation, de l'environnement et du travail (ANSES)

EPIC – Coordinated Action (Contract #006438, SP23-CT-2005-006438); HuGeF (Human Genetics Foundation), Torino, Italy; Cancer Research UK.

EpiLymph – European Commission (grant references QLK4-CT-2000-00422 and FOOD-CT-2006-023103); We thank CERCA programme / Generalitat de Catalunya for institutional support. This work was supported by Spanish Ministry of Economy and Competitiveness - Carlos III Institute of Health cofunded by FEDER funds/European Regional Develpment Fund (ERDF) - a way to build Europe (grant references CIBERESP, PI17/01280, PI20/00288 with the support of the Secretariat for Universities and Research of the Ministry of Business and Knowledge of the Government of Catalonia (2017SGR1085) who had no role in the data collection, analysis or interpretation of the results; the NIH (contract NO1-CO-12400); the Compagnia di San Paolo—Programma Oncologia; the Federal Office for Radiation Protection grants

StSch4261 and StSch4420, the José Carreras Leukemia Foundation grant DJCLS-R12/23, the German Federal Ministry for Education and Research (BMBF-01-EO-1303); the Health Research Board, Ireland and Cancer Research Ireland; Czech Republic supported by MH CZ – DRO (MMCI, 00209805) and **MEYS - NPS I - LO1413**; Fondation de France and Association de Recherche Contre le Cancer.

GEC/Mayo GWAS – National Institutes of Health (CA118444, CA148690, CA92153). Intramural Research Program of the NIH, National Cancer Institute. Veterans Affairs Research Service. Data collection for Duke University was supported by a Leukemia & Lymphoma Society Career Development Award, the Bernstein Family Fund for Leukemia and Lymphoma Research, and the National Institutes of Health (K08CA134919), National Center for Advancing Translational Science (UL1 TR000135).

GELA/LYSA – Institut National du Cancer (INCa, Paris) grant 2008-020

HPFS (Walter C. Willet) – The HPFS was supported in part by National Institutes of Health grants U01 CA167552, R01 CA149445, and R01 CA098122. The authors would like to acknowledge the contribution to this study from central cancer registries supported through the Centers for Disease Control and Prevention's National Program of Cancer Registries (NPCR) and/or the National Cancer Institute's Surveillance, Epidemiology, and End Results (SEER) Program. Central registries may also be supported by state agencies, universities, and cancer centers. Participating central cancer registries include the following: Alabama, Alaska, Arizona, Arkansas, California, Colorado, Connecticut, Delaware, Florida, Georgia, Hawaii, Idaho, Indiana, Iowa, Kentucky, Louisiana, Massachusetts, Maine, Maryland, Michigan, Mississippi, Montana, Nebraska, Nevada, New Hampshire, New Jersey, New Mexico, New York, North Carolina, North Dakota, Ohio, Oklahoma, Oregon, Pennsylvania, Puerto Rico, Rhode Island, Seattle SEER Registry, South Carolina, Tennessee, Texas, Utah, Virginia, West Virginia, Wyoming. The authors assume full responsibility for analyses and interpretation of these data. We would also like to thank the participants and staff of the Health Professionals Follow-up Study for their valuable contributions. The study protocol was approved by the institutional review boards of the Brigham and Women's Hospital and Harvard T.H. Chan School of Public Health, and those of participating registries as required.

IARC HL GWAS - UK component –Leukaemia Research Fund (08031 and 05045 to R.F.J.); Kay Kendall Leukaemia Fund (R.F.J.)

Iowa-Mayo SPORE – NCI Specialized Programs of Research Excellence (SPORE) in Human Cancer (P50 CA97274); National Cancer Institute (P30 CA086862, P30 CA15083); Henry J. Predolin Foundation.

Italian GxE - Italian Association for Cancer Research (AIRC, Investigator Grant 11855) (PC); Fondazione Banco di Sardegna 2010-2012, and Regione Autonoma della Sardegna (LR7 CRP-59812/2012) (MGE).

Mayo Clinic Case-Control – National Institutes of Health (R01 CA92153 and CA200703); National Cancer Institute (P30 CA015083).

MCCS – Melbourne Collaborative Cohort Study (MCCS) cohort recruitment was funded by VicHealth and Cancer Council Victoria. The MCCS was further augmented by Australian National Health and Medical Research Council grants 209057, 396414 and 1074383 and by infrastructure provided by Cancer Council Victoria. Cases and their vital status were ascertained through the Victorian Cancer Registry and the Australian Institute of Health and Welfare, including the National Death Index and the Australian Cancer Database.

MD Anderson – Institutional support to the Center for Translational and Public Health Genomics.

MSKCC – Geoffrey Beene Cancer Research Grant, Lymphoma Foundation (LF5541); Barbara K. Lipman Lymphoma Research Fund (74419); Robert and Kate Niehaus Clinical Cancer Genetics Research Initiative (57470); U01 HG007033; ENCODE; U01 HG007033.

NCI-SEER – Intramural Research Program of the National Cancer Institute, National Institutes of Health, and Public Health Service (N01-PC-65064,N01-PC-67008, N01-PC-67009, N01-PC-67010, N02-PC-71105).

NHS (Meir J. Stampfer) – The NHS was supported in part by National Institutes of Health grants UM1 CA186107, P01 CA87969, R01 CA49449, R01 CA149445,R01 CA098122, and R01 CA134958. The authors would like to acknowledge the contribution to this study from central cancer registries supported through the Centers for Disease Control and Prevention's National Program of Cancer Registries (NPCR) and/or the National Cancer Institute's Surveillance, Epidemiology, and End Results (SEER) Program. Central registries may also be supported by state agencies, universities, and cancer centers. Participating central cancer registries include the following: Alabama, Alaska, Arizona, Arkansas, California, Colorado, Connecticut, Delaware, Florida, Georgia, Hawaii, Idaho, Indiana, Iowa, Kentucky, Louisiana, Massachusetts, Maine, Maryland, Michigan, Mississippi, Montana, Nebraska, Nevada, New Hampshire, New Jersey, New Mexico, New York, North Carolina, North Dakota, Ohio, Oklahoma, Oregon, Pennsylvania, Puerto Rico, Rhode Island, Seattle SEER Registry, South Carolina, Tennessee, Texas, Utah, Virginia, West Virginia, Wyoming. The authors assume full responsibility for analyses and interpretation of these data. We would also like

to thank the participants and staff of the Health Professionals Follow-up Study for their valuable contributions. The study protocol was approved by the institutional review boards of the Brigham and Women's Hospital and Harvard T.H. Chan School of Public Health, and those of participating registries as required.

NSW - NSW was supported by grants from the Australian National Health and Medical Research Council (ID990920), the Cancer Council NSW, and the University of Sydney Faculty of Medicine.

NYU-WHS - National Cancer Institute (R01 CA098661, P30 CA016087); National Institute of Environmental Health Sciences (ES000260).

PLCO - This research was supported by the Intramural Research Program of the National Cancer Institute and by contracts from the Division of Cancer Prevention, National Cancer Institute, NIH, DHHS.

SCALE – Swedish Cancer Society (2009/659). Stockholm County Council (20110209) and the Strategic Research Program in Epidemiology at Karolinska Institutet. Swedish Cancer Society grant (02 6661). National Institutes of Health (5R01 CA69669-02); Plan Denmark.

UCSF2 – The UCSF studies were supported by the NCI, National Institutes of Health, CA1046282 and CA154643. The collection of cancer incidence data used in this study was supported by the California Department of Health Services as part of the statewide cancer reporting program mandated by California Health and Safety Code Section 103885; the National Cancer Institute's Surveillance, Epidemiology, and End Results Program under contract HHSN261201000140C awarded to the Cancer Prevention Institute of California, contract HHSN261201000035C awarded to the University of Southern California, and contract HHSN261201000034C awarded to the Public Health Institute; and the Centers for Disease Control and Prevention's National Program of Cancer Registries, under agreement #1U58 DP000807-01 awarded to the Public Health Institute. The ideas and opinions expressed herein are those of the authors, and endorsement by the State of California, the California Department of Health Services, the National Cancer Institute, or the Centers for Disease Control and Prevention or their contractors and subcontractors is not intended nor should be inferred.

USHER - The **Utah-Sheffield Hematology Epidemiology Research (USHER)** study was supported by funding from the National Cancer Institute (NCI) grant R01 CA134674 (to NJC). Data collection in Utah was supported by the Utah Population Database (UPDB) and Utah Cancer Registry (UCR). The UPDB is supported by HCI (including Huntsman Cancer Foundation, HCF), the University of Utah, and NCI grant

P30 CA2014. The UCR is funded by the NCI's SEER Program, Contract No. HHSN261201800016I, the US Center for Disease Control and Prevention's National Program of Cancer Registries (Cooperative Agreement No. NU58DP006320), the University of Utah, and HCF.
Data collection in Sheffield, UK was supported by funds from Yorkshire Cancer Research and the Sheffield Experimental Cancer Medicine Centre. The USHER study thanks all study participants. In Utah we thank ascertainment, laboratory, and research informatics teams at Huntsman Cancer Institute (HCI), and the Hematology Biobank, Justin Williams, Brandt Jones, Myke Madsen, Brian Avery and Rob Sargent for their important contributions. In Sheffield, we thank the NCRI Haemato-oncology Clinical Studies Group, and colleagues in the North Trent Cancer Network the North Trent Haemato-oncology Database.

WHI – WHI investigators are: *Program Office* - (National Heart, Lung, and Blood Institute, Bethesda, Maryland) Jacques Rossouw, Jared Reis, and Candice Price; *Clinical Coordinating Center* - (Fred Hutchinson Cancer Research Center, Seattle, WA) Garnet Anderson, Ross Prentice, Andrea LaCroix, and Charles Kooperberg; *Steering Committee and Academic Centers* - (University of Alabama at Birmingham) Gretchen Wells; (Albert Einstein College of Medicine) Yasmin Mossavar-Rahmani; (University at Buffalo) Amy Millen; (University at Buffalo) Jean Wactawski-Wende; (Fred Hutchinson Cancer Center) Marian Neuhouser; (Fred Hutchinson Cancer Center) Holly Harris; (University of Massachusetts) Brian Silver; (University of North Carolina) Nora Franceschini; (Stanford Prevention Research Center) Marcia L. Stefanick; (The Ohio State University) Electra Paskett; (Wake Forest University) Mara Vitolins. The WHI program is funded by the National Heart, Lung, and Blood Institute, National Institutes of Health, U.S. Department of Health and Human Services through 75N92021D00001, 75N92021D00002, 75N92021D00003, 75N92021D00004, 75N92021D00005.

YALE – National Cancer Institute (CA62006); National Cancer Institute (CA165923).
